# Reduced Graphene Oxide Embedded with ZnS Nanoparticles as Catalytic Cathodic Material for Li-S Batteries

**DOI:** 10.3390/nano13142149

**Published:** 2023-07-24

**Authors:** Roberto Colombo, Daniele Versaci, Julia Amici, Federico Bella, Maria Laura Para, Nadia Garino, Marco Laurenti, Silvia Bodoardo, Carlotta Francia

**Affiliations:** Department of Applied Science and Technology (DISAT), Politecnico di Torino, C.so Duca degli Abruzzi 24, 10129 Torino, Italy; roberto.colombo@polito.it (R.C.); daniele.versaci@polito.it (D.V.); julia.amici@polito.it (J.A.); federico.bella@polito.it (F.B.); maria.para@polito.it (M.L.P.); nadia.garino@polito.it (N.G.); marco.laurenti@polito.it (M.L.); silvia.bodoardo@polito.it (S.B.)

**Keywords:** reduced graphene oxide, lithium-sulfur battery, catalytic material, zinc-sulfide nanoparticles, microwave synthesis

## Abstract

Lithium-sulfur technology is a strong candidate for the future generation of batteries due to its high specific capacity (1675 mAh g${}^{-1}$), low cost, and environmental impact. In this work, we propose a facile and solvent-free microwave synthesis for a composite material based on doped (sulfur and nitrogen) reduced graphene oxide embedded with zinc sulfide nanoparticles (SN-rGO/ZnS) to improve the battery performance. The chemical-physical characterization (XRD, XPS, FESEM, TGA) confirmed the effectiveness of the microwave approach in synthesizing the composite materials and their ability to be loaded with sulfur. The materials were then thoroughly characterized from an electrochemical point of view (cyclic voltammetry, galvanostatic cycling, Tafel plot, electrochemical impedance spectroscopy, and Li${}_{2}$S deposition test); the SN-rGO/ZnS/S${}_{8}$ cathode showed a strong affinity towards polysulfides, thus reducing their loss by diffusion and improving redox kinetics, allowing for faster LiPSs conversion. In terms of performance, the composite-based cathode increased the specific capacity at high rate (1 C) from 517 to 648 mAh g${}^{-1}$. At the same time, more stable behavior was observed at 0.5 C with capacity retention at the 750th cycle, where it was raised from 32.5% to 48.2%, thus confirming the beneficial effect of the heteroatomic doping process and the presence of zinc sulfide nanoparticles.

## 1. Introduction

In the past two decades, the demand for energy produced through renewable sources has continuously increased, which has limited fossil fuel utilization and related CO${}_{2}$ emissions [1]. Therefore, the development of safer energy storage systems with better performances is at the center of scientific research. Nowadays, the energy storage market is dominated by lithium-ion batteries, which present many advantages such as high capacity and long cycle life [2,3,4]. However, the theoretical capacity of this battery is close to being reached [5]. Future alternative technologies focus on various drivers such as low cost materials, high energy density, suitable battery size, highly reversible processes, and long cycle life, which translates into very efficient technology. In this respect, some alternatives technologies, such as the Zn-ion battery [6,7], the aqueous ammonium-ion batteries (AAIBs) [8], or the lithium-selenium battery [9] are under investigation. As far as Li-based batteries are concerned, the lithium-sulfur battery (LSB) is moving closer to commercialization as it exhibits several advantages such as high theoretical specific capacity (1675 mAh g${}^{-1}$), high energy density (2500 Wh kg${}^{-1}$), environmental friendliness, and low cost [10,11,12]. However, some challenges must be overcome to develop the LSBs on the industrial scale. Firstly, the insulating nature of sulfur and its discharge products (Li${}_{2}$S${}_{2}$ and Li${}_{2}$S) limits the utilization of active materials, leading to slower reaction kinetics and poor rate performance. Secondly, the volume change between S${}_{8}$ and Li${}_{2}$S can generate cracks inside the cathode, thus decreasing the cycling performances of the cell [13,14]. The working principle of the LSBs is based on the reversible reaction:

(1) $$2Li^{+}+2e^{-}+xS⇌Li_{2}S_{X}$$

When the cell is discharged, the redox reaction forms lithium polysulfides (LiPSs) intermediates, consisting of Li${}_{2}$S${}_{x}$ chains, where x = 1 at the end of the discharge process [15]. The pivotal complication of LSBs is the so-called shuttle effect: this phenomenon consists of the migration of LiPSs from the cathodic to the anodic side during the cycling of the cell, and it causes irreversible loss of active material, a consistent decrease in the specific capacity, and poor coulombic efficiency [16,17]. Up to now, massive efforts have been devoted to mitigating the extent of such issue. Recently, the utilization of gel polymer electrolytes (GPEs), which are able to suppress the shuttle effect and reduce the risk of leakage and flammability, has also been investigated [18]. One approach consists of using carbon-based materials such as graphene and carbon nanotube as hosts for sulfur to restrain the shuttle effect by physical confinement of lithium polysulfides (LiPSs) and to improve the conductivity [19,20]. However, carbon-based materials present a non-polar nature which hinders a strong chemical interaction with the polar LiPSs intermediates, hence the researchers have drawn their attention to polar materials such as metal oxides and metal sulfides as agents to actively interact with LiPSs and reduce their diffusion in the electrolyte, thus relieving the shuttle effect [21,22,23]. An interesting approach consists of combining carbonaceous materials such as hierarchically porous and microporous carbons [24,25], N-doped carbon nanofibers [26], and graphene-based porous carbon [27] with different materials such as Co and CoSe nanoparticles [28,29] or metal oxides/sulfides, in order to simultaneously exploit the excellent electronic conductivity of the former and the high chemical affinity with the polysulfides of the latter. In particular, metal sulfides have been investigated as cathode materials for Li-S batteries due to their easy preparation, high electrochemical activity and redox chemistry [30,31]. Moreover, it has been recently reported that some of these materials, such as VS${}_{2}$, TiS${}_{2}$, CoS${}_{2}$ may act as catalysts to promote the oxidation of Li${}_{2}$S [32]. Thus, the employment of sulfide-based cathodes is an auspicious method to reduce the shuttle effect and attain high sulfur utilization and significantly enhance the properties of Li–S batteries [33]. In this regard, Zhang et al. [34] designed a composite cathode where the ZnS nanoparticles are used as a booster for the conversion of LiPSs, while Liu et al. [35] introduced an interlayer embedded with ZnS nanoparticles to trap the LiPSs and reduce the shuttle effect.

In this work, we prepared an innovative composite material based on a nitrogen-sulfur-doped graphene matrix on which zinc sulfide nanoparticles were directly nucleated in a one-step microwave synthesis. The combination of the two materials turns out to be a winning strategy for obtaining a cathode material suitable for lithium-sulfur batteries: the graphitic network can provide good electronic conductivity for the conversion of the sulfur, while the graphene doping and the presence of ZnS nanoparticles guarantee a strong interaction and catalytic activity towards LiPSs, thus alleviating the shuttle effect.

## 2. Materials and Methods

### 2.1. Materials and Reagents

The graphene oxide (GO) was purchased from Cheaptubes, H${}_{2}$N–C(=S)–NH${}_{2}$, LiNO${}_{3}$ and (CH${}_{3}$COO)${}_{2}$Zn·2H${}_{2}$O were purchased by Sigma Aldrich (St. Louis, MO, USA), Ketjenblack (KjB EC-300J) and the poly(vinylidene difluoride) (PVdF) were provided by AkzoNobel and Solvay, respectively.

### 2.2. Materials Preparation

#### 2.2.1. SN-rGO and SN-rGO/ZnS Synthesis

All the chemicals were used as purchased without further purification. The sulfur-nitrogen doped rGO (labelled as SN-rGO) and the sulfur-nitrogen doped rGO embedded with ZnS nanoparticles, labelled as SN-rGO/ZnS), were prepared by following the synthesis procedure reported below. With regards to the SN-rGO sample, in a microwave 100 mL Teflon reactor equipped with pressure and temperature probes (Milestone FlexyWave, Milestone Inc., Shelton, CT, USA), 105 mg of GO (Cheap Tubes Inc., Cambridgeport, VT, USA) were added to 50 mL DI water and sonicated (Elmasonic P 30H) for 30 min. After that, 71 mg of H${}_{2}$N–C(=S)–NH${}_{2}$ were added and the precursors mixture was sonicated again for about 30 min. The resulting homogeneous slurry was irradiated for 15 min at 200 °C (900 W maximum) with a microwave heating ramp of 2 min to reach the desired temperature. The maximum pressure reached during the process was 15 bar. The reactor was then cooled down to RT and the resultant suspension was then collected in small vessels, washed with DI water, and freeze-dried (Lio 5P, 5Pascal, Trezzano sul Naviglio Milano, Italy) until all the water was removed. The SN-rGO/ZnS synthesis was carried out following a similar procedure: 105 mg of GO and 219 mg of (CH${}_{3}$COO)${}_{2}$Zn·2H${}_{2}$O were dispersed in 35 mL and 15 mL of DI water, respectively. After 30 min of sonication, the two solutions were mixed, and 142 mg of thiourea were added. The microwave synthesis parameters remained the same as used for the SN-rGO sample.

#### 2.2.2. Preparation of SN-rGO/S${}_{8}$ and SN-rGO/ZnS/S${}_{8}$ Composites

The same procedure was followed for both SN-rGO and SN-rGO/ZnS samples. Pure sulfur (≥99.5% Sigma Aldrich) and the SN-rGO (and SN-rGO/ZnS) powders were gently mixed using a pestle and mortar in a weight ratio of 3:1. The mixture was then transferred to a furnace tube under argon stream, where the material was heated with a hitting rate of 2.5 °C/min to 155 °C for 12 h, in order to obtain the infiltration of the sulfur in the carbonaceous material. The melt-infused composites, referred as SN-rGO/S${}_{8}$ and SN-rGO/ZnS/S${}_{8}$, were subsequently finely crushed into powder to make them suitable for the cathode preparation.

### 2.3. Preparation of the SN-rGO/S${}_{8}$, SN-rGO/ZnS/S${}_{8}$ Cathodes and SN-rGO, SN-rGO/ZnS, KjB Electrodes

The cathodes were obtained through the solvent tape casting method. The SN-rGO/S${}_{8}$ and SN-rGO/ZnS/S${}_{8}$ were used as active materials, Ketjenblack, as conductive carbon additive and (PVdF, 8 wt.% in N-methyl-2-pyrrolidinone solution) as polymeric binder. The electrode composition was set up 80:10:10 wt.%, respectively, for (SN-rGO/S${}_{8}$:KjB:PVdF) or (SN-rGO/ZnS/S${}_{8}$:KjB:PVdF) in all preparations. The mixture of these components was softly ball-milled to obtain a homogeneous slurry and mechanically deposited on the aluminum current collector by the doctor blade technique. The blade was adjusted for a 200 µm deposition using an automatic film applicator (Sheen-AB4120) with a speed of 10 mm s${}^{-1}$. After the slurry deposition, the coated aluminum foil was dried at 50 °C in the air; subsequently, disks of 1.76 cm${}^{2}$ were punched out and vacuum dried at 40 °C (in a Büchi Glass Oven B-585) for 4 h then transferred into an Argon filled dry glove-box (MBraum Labstar, H${}_{2}$O and O${}_{2}$ content < 1 ppm) for cell assembly. The active material loading (referred to the S${}_{8}$) for both the SN-rGO/S${}_{8}$ and SN-rGO/ZnS/S${}_{8}$-based cathodes was between 1 and 1.3 mg cm${}^{-2}$. The electrodes used for the catalytical test (Tafel, symmetrical cyclic voltammetry and Li${}_{2}$S deposition) were produced by casting a slurry of 80:10:10 wt % (SN-rGO:KjB:PVdF), (SN-rGO/ZnS:KjB:PVdF) and 90:10 wt% (KjB:PVdF) on carbon paper (CB, GDL 39BB SGL Carbon, Wiesbaden, Germany) with a thickness of 200 µm. The as-prepared electrodes do not contain any sulfur. The cutting and drying procedure was the same as the one used for the cathodes.

### 2.4. Material Characterization

XRD analysis was carried out by a PANalytical X’Pert (Cu Ka radiation) diffractometer. Data were collected with a 2D solid state detector (PIXcel) from 10 to 80°2ϑ with a step size of 0.026°2ϑ and a wavelength of 1.54187 Å. The Brunauer-Emmett-Teller specific surface area (SSA) was determined by nitrogen physisorption at 77 °K using a Micrometrics ASAP 2020 instrument. The specific surface area was calculated with the BET model in the relative pressure range of 0.07–0.30 by assuming 0.162 nm${}^{2}$/mol as the molecular area of nitrogen. X-ray photoelectron spectroscopy (XPS) measurements were carried out using a PHI Model 5000 electron spectrometer equipped with an aluminum anode (1486 eV) monochromatic source, with a power of 25.0 W, and high-resolution scan with 11.75 eV pass energy. The instrument typically operates at pressures below 5 × 10${}^{-8}$ mbar. Field emission scanning electron microscopy (FESEM) analysis was carried out by Zeiss SUPRA™ 40 with Gemini column and Schottky field emission tip (tungsten at 1800 K). Acquisitions were made at an acceleration voltage of 3 kV and a working distance between 2.1 and 8.5 mm, with magnification up to 1000 kX. Transmission electron microscopy (TEM) analysis was performed by high-resolution JEOL 300 kV. Thermogravimetric analysis was performed on a TG 209 F Tarsus (Netzsch, Selb, Germany) instrument by heating the samples at 10 °C min${}^{-1}$ from room temperature to 800 °C and 1000 °C in air, to evaluate the sulfur content inside the composite material.

The UV-vis absorption spectra were detected by a UV–vis spectrophotometer (Hitachi U-5100 Spectrophotometer, Tokyo, Japan) within the spectral range of 300–550 nm.

### 2.5. Electrochemical Measurement

To measure the electrochemical properties of the SN-rGO/S${}_{8}$ and SN-rGO/ZnS/S${}_{8}$ materials, electrodes based on these materials were assembled in CR2032 coin-type cells with lithium disk (Chemetall Foote Corporation, Ø16 mm) as counter electrode and PP polymeric membrane (Celgard 2500, 25 μm thickness, Ø19 mm) as separator.

The electrolyte was a solution of 1,2-dimethoxyethane (DME) and 1,3-dioxolane (DIOX) 1:1 (*v*/*v*) with 1.0 M lithium bis(trifluoromethanesulfonyl)imide (CF${}_{3}$SO${}_{2}$NLiSO${}_{2}$CF${}_{3}$, LiTFSI) and 0.25 M lithium nitrate (LiNO${}_{3}$, ≥99.9%). The amount of electrolyte was calculated based on the sulfur content in each cathode, always maintaining a ratio of 10–11 μL of electrolyte per mg of sulfur.

In order to evaluate the cycling performances of the cathodes, the cells were tested employing galvanostatic discharge-charge cycling (GC) using an Arbin LBT-21084 battery tester at room temperature. Galvanostatic discharge-charge tests were carried out in the potential interval 1.85–2.6 V vs. Li/Li${}^{+}$ at different current rates. The C-rate was calculated using the theoretical capacity of sulfur (1675 mAh g${}^{-1}$). The cyclic voltammetry (CV) was performed in the potential range between 1.7 and 2.8 V vs. Li/Li${}^{+}$ at 0.05, 0.1 mVs${}^{-1}$ and between 1.7 and 3.0 V vs. Li/Li${}^{+}$ at 0.2, 0.3, 0.4 and 0.5 mVs${}^{-1}$. The CV test at different scan rates was carried out to evaluate the diffusion coefficient of Li${}^{+}$ in the electrode using the Randles-Sevcik equation:

(2) $$i_{p}=\left(2.687\times 10^{5}\right)n^{\frac{3}{2}}AC_{Li^{+}}D^{\frac{1}{2}}$$

where the constant term 2.687 × 10${}^{5}$ has the unit of [C·mol${}^{-1}$·V${}^{-\frac{1}{2}}$ ], *n* is the number of electrons transferred in a redox cycle, *A* is the electrode surface area [cm${}^{2}$], C${}_{L}i^{+}$ is the concentration of Li${}^{+}$ inside the cathodic material [mol·cm${}^{-3}$] and *D* is the lithium-ion diffusion coefficient [cm${}^{2}$·s${}^{-1}$]. Electrochemical Impedance Spectroscopy tests (EIS) were performed with Bio-Logic^®^VSP-3e multichannel potentiostat equipped with impedance modules. The spectra were recorded in the frequency range of 100 kHz to 10 mHz, with an excitation potential of 5 mV and 10 points per decade. The EIS spectra were collected at the end of the anodic scan (2.8 V vs. Li/Li${}^{+}$ for the scan rate performed at 0.05 and 0.1 mVs${}^{-1}$, and 3.0 V vs. Li/Li${}^{+}$ for the 0.2, 0.3, 0.4 and 0.5 mVs${}^{-1}$), after 30 min of rest, at each different scan rate that was performed.

Li${}_{2}$S${}_{8}$ symmetric cells were assembled in an argon-filled glove box to study the kinetic behavior of the SN-rGO/S${}_{8}$ and SN-rGO/ZnS/S${}_{8}$ materials [36]. In particular, two identical electrodes were assembled into a CR2032 coin cell with a Celgard 2500 membrane as a separator, while 50 µL of 1,2-dimethoxyethane (DME) and 1,3-dioxolane (DIOX) 1:1 (*v*/*v*) Li${}_{2}$S${}_{8}$ 0.125 M solution was used as catholyte. The symmetrical CV test was repeated with a blank solution of 1,2-dimethoxyethane (DME) and 1,3-dioxolane (DIOX) 1:1 (*v*/*v*).

The Tafel and the Li${}_{2}S$ precipitation tests were carried out on CR2032 coin cells composed of the carbon fiber electrodes of SN-rGO, SN-rGO/ZnS, and KjB vs. lithium disk. For the former, performed by mean of two linear sweep voltammetry at 0.005 mVs${}^{-1}$ from the open circuit voltage of the cell to ±30 mV, after one hour of rest, 50 µL of 1,2-dimethoxyethane (DME) and 1,3-dioxolane (DIOX) 1:1 (*v*/*v*) Li${}_{2}$S${}_{8}$ 6.25 mM solution was used as catholyte, while for the latter the concentration of Li${}_{2}$S${}_{8}$ was 25 mM.

## 3. Results and Discussion

### 3.1. Morphological Characterization

**Figure 1:**
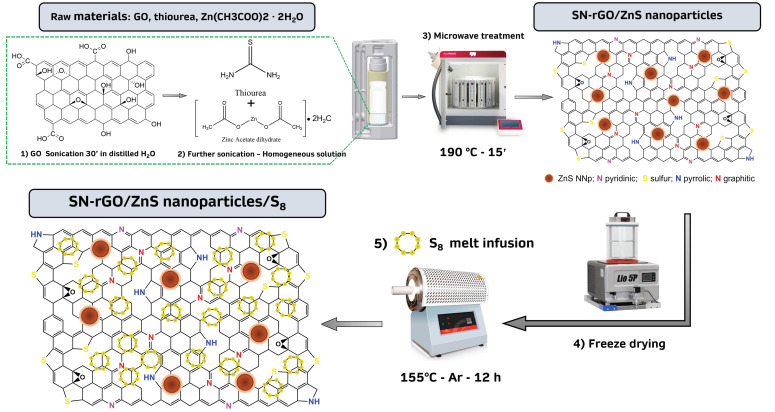
Scheme of the microwave-assisted synthesis of the SN-rGO, SN-rGO/ZnS, SN-rGO/S${}_{8}$ and SN-rGO/ZnS/$S_{8}$ samples.

The microwave synthesis approach that was used in this work to obtain the SN-rGO and the SN-rGO-ZnS is illustrated in Figure 1. Initially, the aqueous dispersion of GO was sonicated for 30 min to increase the number of negative charges on its surface to attract the Zn${}^{2+}$ ions via electrostatic interaction and to generate defective sites [37,38]. With the increment of temperature, the thermal reduction of GO partially takes place but is not completed. When the value of T reaches 200 °C, thiourea decomposes into highly reactive N/S-rich species such as H${}_{2}$S, NH${}_{3}$, and CS${}_{2}$, which are highly prone to react with the aforementioned defective site generated by the removal of oxygen-containing groups of GO, thus concluding the reduction and doping process [39,40]. Concurrently with the described process, the nucleation and growth of ZnS nanoparticles take place on the surface of rGO thanks to the coordination of S${}^{2-}$ ions, originated by CS${}_{2}$, with Zn${}^{2+}$ ions. It is worth to remark the fact that the whole procedure to carry out the synthesis is extremely optimized in terms of time and steps needed: thiourea is used both as a precursor for GO doping and as a source of sulfur for the generation of ZnS nanoparticles, while the microwave heating process is orders of magnitude faster than the classic hydrothermal route [41].

**Figure 2:**
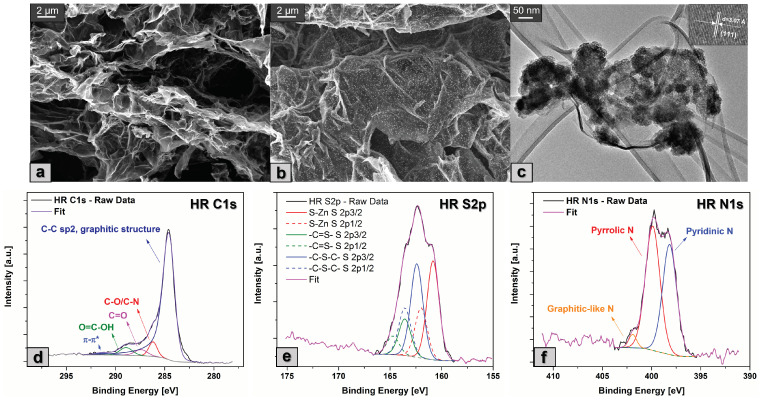
(**a**) FESEM micrograph of SN-rGO sample; (**b**) FESEM micrograph of SN-rGO/ZnS sample, where the ZnS particles are clearly visible on the surface of the doped rGO; (**c**) TEM micrograph of SN-rGO/ZnS, with particular focus on the ZnS nanoparticles and their crystalline fringes and interplanar (111) distance in the inlet in the upper right corner; (**d**) XPS C1s high-resolution spectra; (**e**) XPS S2p high-resolution spectra; and (**f**) XPS N1s high-resolution spectra of the SN-rGO/ZnS sample.

The morphological characterization of SN-rGO and SN-rGO/ZnS was performed by FESEM technique, as reported in Figure 2a and b, respectively. In particular, it is possible to observe that both samples exhibit the typical exfoliated structure of rGO, with the evident presence of nanoparticles on the carbonaceous surface for the sample that was synthesized with the presence of (CH${}_{3}$COO)${}_{2}$Zn·2H${}_{2}$O. The average dimensions of these nanoparticles lie around 80 nm, as reported in Figure S1, while their chemical composition was investigated by means of EDS analysis in Figure S2. Notably, when the elemental analysis of the nanoparticles is compared with the one of a larger area of the same sample, as shown in Figure S3, it is clear that they consist of Zn and S, since the weight and atomic percentages of these elements are considerably higher than those of the others. The morphology of the SN-rGO/ZnS sample was further characterized by TEM, and the resulting micrograph is depicted in Figure 2c. Essentially, this analysis highlighted the crystallinity of the ZnS nanoparticles, in fact it was possible to evaluate the distance between the (111) planes to be around 3.07 Å, in agreement with the existing literature [42,43]. Moreover, from Figure 2c it is possible to notice that the ZnS nanoparticles are, in reality, composed of agglomerates of much smaller primary particles.

The functional groups and surface chemical composition of the SN-rGO and SN-rGO/ZnS were investigated by means of X-ray photoelectron spectroscopy (XPS) as shown in Figure S4 and Figure 2. The survey spectra of both samples (Figure S4) exhibit S2p, C1s, and N1s peaks at about 164, 284, and 400 eV, respectively, while the additional peak at around 1021.4 eV of the Zn2p, was detected only in the case of SN-rGO/ZnS sample. In particular, the initial conclusions that can be drawn from the survey spectra are the following: firstly, the incorporation of nitrogen and sulfur in the graphene networks is confirmed by the presence of their relative signals, thus corroborating the hypothesis of the heteroatoms doping; secondly, the presence of thiourea appears to be beneficial for the reduction degree of the GO, since the C/O ratio of our samples is 3.01 in the case of SN-rGO and 4.62 for SN-rGO/ZnS, well above the typical value reported for GO [44,45]. The nitrogen content in the SN-rGO and SN-rGO/ZnS reaches, respectively, 8.9 at.% and 7.4 at.%, while the sulfur doping is estimated to be 7.25 at.% for the former and 6.5 at.% for the latter, as shown in Table S1. The chemical environment of C, N, S and Zn atoms in our samples were investigated by high-resolution C1s, N1s, S2p, and Zn2p spectra, reported in Figure 2 and Figure S5 for the SN-rGO and Figure S6 for the SN-rGO/ZnS sample. In particular, the deconvolution of the C1s spectrum of both SN-rGO and SN-rGO/ZnS (Figure S5a and Figure 2d) highlights the reduction of the intensity of the typical oxygen-containing functionalities of GO such as C-O/C=O and O=C-OH with the introduction of N and S into the carbon network [46]. The high-resolution S2p peak of the SN-rGO/ZnS was fitted with three different peaks associated with their relative doublets (Figure 2e). The peak at 160.8 eV was attributed to the presence of the Zn-S bond, thus confirming the formation of ZnS nanoparticles, while the peaks at 162.4 and 163.6 eV were ascribed to the thiophenic -C-S-C- and the conjugated -C=S- bond, respectively. The formation of this kind of bond is most likely due to the reaction between oxygen-containing groups in GO and the H${}_{2}$S/CS${}_{2}$ developed during the reduction process. The S2p spectrum of the SN-rGO sample, reported in Figure S5b, exhibits a large peak at around 164.3 eV, which was attributed to the cumulative signal of S2p${}_{3/2}$ and S2p${}_{1/2}$ of -C-S-C- and -C=S- bonds, and it is not present at the peak at 160.8 eV proving that the nanoparticles of ZnS are formed in the composite SN-rGO/ZnS (Figure 2e). The other two peaks at 162 and 168.5 eV were ascribed to the presence of sulfide and sulfate -SO${}_{x}$ groups that can be present at the edges of graphene [47]. The N1s spectra of both SN-rGO and SN-rGO/ZnS were deconvoluted with three peaks, as shown in Figure S5c and Figure 2f: the nitrogen appears to be mostly present as pyridinic and pyrrolic N in both samples, with a lower content of graphitic-like N. In particular, it is worth noting that the amount of pyridinic N is almost double in the case of SN-rGO/ZnS compared to SN-rGO. This difference is not insignificant when considering our application since the pyridinic N is often reported as a preferential adsorption site of LiPSs [48,49,50]. The quantification of the XPS data for the two samples is reported in Table S2.

**Figure 3:**
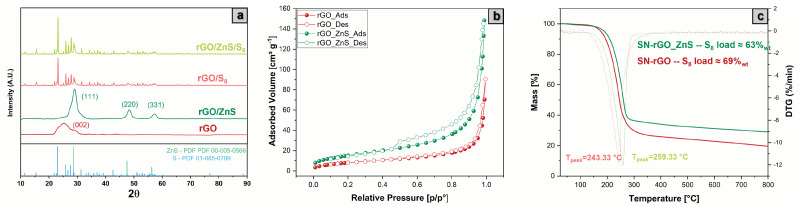
(**a**) XRD spectra of the SN-rGO, SN-rGO/ZnS, SN-rGO/S${}_{8}$ and SN-rGO/ZnS/S${}_{8}$ samples from bottom to top, respectively (**b**) N${}_{2}$ adsorption-desorption isotherms of SN-rGO and SN-rGO/ZnS (**c**) TGA of SN-rGO/S${}_{8}$ and SN-rGO/ZnS/S${}_{8}$ samples in air from 25 °C to 800 °C.

The characterization of the pristine SN-rGO and SN-rGO/ZnS was completed utilizing XRD, BET and TGA techniques, which were also used to study the two materials after they had been loaded with sulfur in the tubular oven (Figure 1). The infiltrated samples are referred as SN-rGO/S${}_{8}$ and SN-rGO/ZnS/S${}_{8}$. The XRD spectra of the SN-rGO and SN-rGO/ZnS, reported in Figure 3a do not present any signal at 2θ = 10.6 which is ascribed to the (001) lattice plane corresponding to a d spacing of 0.83 nm in GO, thus certifying the reduction of GO during the microwave synthesis. In the SN-rGO sample, thanks to the removal of oxygen-containing groups, a broad diffraction peak (002) between 21° and 30° appears, corresponding to a d-spacing of 0.38 nm [51]. Finally, in the SN-rGO/ZnS spectra is evident the presence of a crystalline phase that was identified by the typical positions at 28.5°, 47.6° and 56.3° of the (111), (220), and (311) lattice plans of ZnS, respectively. The XRD spectra of the two rGO infiltrated with sulfur, reported in the upper part of Figure 3a, are very similar to each other due to the clear presence of sulfur patterns that cover the peaks of the two pristine materials.

The nitrogen adsorption-desorption analysis of SN-rGO and SN-rGO/ZnS, shown in Figure 3b, reveal some differences: the curve of the former resembles a reversible Type II isotherm, which indicates monolayer coverage and multilayer adsorption in non-porous adsorbents, while the isotherm of the latter is characterized by a hysteresis loop of Type H3, which could be ascribed to the presence of slit-shaped pores resulting from the overlap of several rGO layers separated by the ZnS nanoparticles [52,53]. These results are in agreement with the morphology exhibited by the two samples in Figure 2a,b. The isotherms of the two samples after being infiltrated with sulfur (Figure S7a,b) exhibit a reversible Type II behavior with no evidence of porosity, most likely due to the insertion of S${}_{8}$ in the pores. The specific surface area of SN-rGO, SN-rGO/ZnS, SN-rGO/S${}_{8}$ and SN-rGO/ZnS/S${}_{8}$ were calculated to be 30.6 m${}^{2}$ g${}^{-1}$, 56.9 m${}^{2}$ g${}^{-1}$, 7.9 m${}^{2}$ g${}^{-1}$ and 8.8 m${}^{2}$ g${}^{-1}$ using Brunauer– Emmett–Teller (BET) model. Therefore, it is clear that the presence of ZnS nanoparticles boosts the surface area by nearly twofold, and this feature is also desirable to increase the number of available sites for the LiPSs adsorption. Similarly, after the S infiltration for both composite the surface specific is comparable, which means that the pores induced by the presence of nanoparticles are blocked after S${}_{8}$ loading. Finally, the thermogravimetric analysis (TGA) was carried out on all prepared samples in order to evaluate the weight percentage of ZnS nanoparticles in the SN-rGO/ZnS and the sulfur loading in the two infiltrated samples. In particular, Figure S8 reports the TGA results of SN-rGO and SN-rGO/ZnS, which point out the presence of a large residue (48.11 wt.%) in the case of SN-rGO/ZnS. From this data, it is possible to estimate the weight percentage of ZnS to be around 57.6%${}_{wt}$, considering the ratio between the molecular weight of ZnS and the ZnO, since at high temperatures, the ZnS is oxidized in the latter [54]. The TGA curves of the composite materials after the melt infusion step, depicted in Figure 3c, are characterized by the same trend over temperature, with the SN-rGO and SN-rGO/ZnS samples being able to include around 69%${}_{wt}$ and 63%${}_{wt}$ of sulfur inside their structure, respectively. This difference could be ascribed to the different pore sizes of the two materials that were previously evidenced by the nitrogen adsorption-desorption analysis.

### 3.2. Electrochemical Characterization

**Figure 4:**
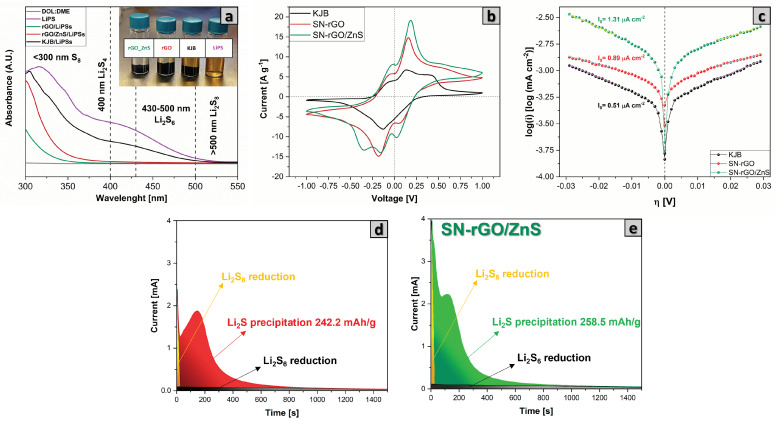
(**a**) UV-VIS analysis of the pure DOL:DME Li${}_{2}$S${}_{8}$ 0.5 mM solution and supernatants of the same solution after being in contact with SN-rGO and SN-rGO/ZnS; (**b**) cyclic voltammetry of the symmetrical cells (both electrodes of KjB, SN-rGO and SN-rGO/ZnS) performed at 5 mV s${}^{-1}$; (**c**) Tafel plots of the Li${}_{2}$S${}_{8}$ solution redox on KjB, SN-rGO and SN-rGO/ZnS materials, derived from positive and negative LSV scans; (**d**) current transients obtained during the potentiostatic step of the Li${}_{2}$S deposition test the for SN-rGO; and (**e**) for the SN-rGO/ZnS.

Since one of the fundamental characteristics of cathode material in lithium-sulfur batteries is to have a good interaction and enhanced catalytic activity to accomplish a faster kinetic reaction of conversion of LiPSs to reduce the shuttle effect, we focused the first electrochemical tests on investigating our samples in this regard. In particular, the adsorption capability of our materials (pure KjB, SN-rGO, SN-rGO/ZnS) towards LiPSs was evaluated by dipping 30 mg of powders in 4 mL of DME:DIOX 1:1 mixture Li${}_{2}$S${}_{8}$ 0.5 mM; the solution was stirred and allowed to stand overnight. The different samples are depicted in the upper right corner of Figure 4a, from which it is possible to notice that the color of the solution essentially remains unchanged in the case of the KjB, thus demonstrating its negligible adsorption capability. In contrast, the SN-rGO sample can considerably bleach the solution, even though complete transparency of the supernatant was achieved only in the case of the SN-rGO/ZnS sample. In order to better evaluate these interactions, we performed the ultraviolet-visible absorption spectroscopy, whose results are reported in Figure 4a. A pure mixture of DME:DIOX 1:1 was used to create a baseline, while the pure 0.5 mM Li${}_{2}$S${}_{8}$ 0.5 mM solution served as a reference to individuate the typical peaks ascribed to the different Li${}_{2}$S${}_{x}$ species, which are visible in the purple spectra of Figure 4a. After the adsorption test, it can be clearly seen that the intensity of the LiPSs peaks dramatically decreases in the case of SN-rGO and is almost zeroed in the SN-rGO/ZnS spectrum. These results confirm the ability of our samples to capture LiPSs and agree with the existing literature. In fact, the simultaneous introduction of nitrogen and sulfur into the graphene structure allows to generate particularly effective sites in the adsorption of LiPSs. More specifically, when a pyrrolic or pyridinic N is found adjacent to the thionic sulfur, the binding energy between graphene and LiPSs species increases considerably, making the SN-rGO an efficient material in trapping LiPSs [45,55,56]. In addition to this, the presence of ZnS nanoparticles appears to be beneficial in further increasing the interaction with LiPSs since the adsorption energies predicted by DFT calculations by Razaq [37] and Xu [56] of Li${}_{2}$S${}_{4}$ on the 100, 110, and 111 facets of ZnS are significantly higher than those predicted in the case of graphene.

In order to verify the role played by the doping process and ZnS nanoparticles in the kinetic reaction of LiPSs conversion, we performed cyclic voltammetry (CV) tests in the voltage window −1 ∼ 1 V of symmetrical cells with both a catholyte Li${}_{2}$S${}_{8}$ 0.125 M and Standard electrolyte (see Section 2.1), as reported in Figure 4b and Figure S9, respectively. Unsurprisingly, the CV of the Li${}_{2}$S${}_{8}$ free symmetrical cells (Figure S9a) are characterized by an almost flat voltammogram, with the annexed capacity due to the capacitive current. On the contrary, when the Li${}_{2}$S${}_{8}$ is introduced in the formulation of the electrolyte, both the SN-rGO and SN-rGO/ZnS exhibit a much stronger current response, which consequently appears to be dominated by the typical conversion reactions of Li-S battery rather than double-layer capacitance. In particular, the SN-rGO/ZnS sample shows good reversibility and facile polysulfide conversion considering the distinct peaks at −0.092, 0.087, 0.342 V in reduction and 0.079, −0.086, −0.291 V in oxidation which are visible in Figure 4 and even more in the scan performed at 1 mV${}^{-1}$, reported in Figure S9b. More specifically, the peaks in the cathodic scan are indicative of the step-by-step reduction from original Li${}_{2}$S${}_{8}$ to insoluble Li${}_{2}$S on the working electrode and oxidation of Li${}_{2}$S${}_{8}$ on the counter electrode (see Table S3 in supporting information for the Equations (S1)–(S9) describing the reaction path during the scan) [57,58]. Furthermore, the current response in the symmetrical CVs turns out to be in the order SN-rGO/ZnS > SN-rGO > KjB, thus corroborating the hypothesis that the kinetic of the conversion of polysulfides is primarily increased by the ZnS nanoparticles but also partially by the heteroatomic doping process of graphene by N and S.

The redox kinetics of our samples during the discharge and charge process was investigated by current exchange measurements using linear sweep voltammetry (LSV) with a Li${}_{2}$S${}_{8}$ catholyte solution in coin cell configuration. In particular, Figure 4c depicts the Tafel plot of KjB, SN-rGO, and SN-rGO/ZnS for the cathodic and anodic reactions. What can be easily deduced from this graph is that the lithiation and delithiation overpotentials of S have been substantially decreased by the SN doping process but even more by the presence of ZnS particles on the graphene sheets [59]. Following the procedure reported by Nazar [60], the exchange current densities, which give an idea of how fast is the electron transfer rate, were derived from the Tafel plot. The calculated values (0.51 μA cmg${}^{-2}$ for KjB, 0.89 μA cmg${}^{-2}$ for SN-rGO and 1.31 μA cmg${}^{-2}$ for SN-rGO/Zns) highlight the faster charge transfer kinetics induced by the ZnS nanoparticles.

Another essential aspect that must be considered in lithium-sulfur batteries is the liquid-to-solid conversion process of long polysulphides in Li${}_{2}$S. In particular, considering the insulating nature of Li${}_{2}$S, it is crucial to make the deposition process of solid Li${}_{2}$S as controllable as possible. In order to study how the lithium sulfide deposition process took place on our different samples, we carried out an experiment where the cells (see Section 2.4 for the cell assembly) were discharged with a current of 100 uA up to 2.15 V and then a potentiostatic step at 2.02 V was applied until the recorded current dropped to zero. The results of the Li${}_{2}$S deposition test for the SN-rGO and SN-rGO/ZnS samples are shown in Figure 4d and e, respectively. Qualitatively, the formation of a non-faradaic double-layer plus the reduction of long-chain polysulfides to mid-chain polysulfides causes the initial current drop of the current-time response [61]. The following part of the curve reflects a nucleation and growth process. Initially, during the nucleation process, the current growth indicates an increase in the electroactive area due to the rise in the number of Li${}_{2}$S nucleation centers or the size growth of each nucleus precipitated on the electrode surface [62]. Afterwards, during the growth phase the current drops due to coalescence between neighboring nuclei centers and the overlap of the adjacent hemispherical diffusion zone developed by the sparsely distributed nuclei to an infinite linear concentration gradient [63]. Going into more detail, it can be seen that, on the basis of Faraday law, the Li${}_{2}$S deposition capacities on the SN-rGO and SN-rGO/ZnS are 242.2 and 258.5 mAh g${}^{-1}$, respectively, indicating that the presence of ZnS nanoparticles provides more active sites for the nucleation and growth of Li${}_{2}$S.

To exhaustively study how the morphology of our samples and the ZnS nanoparticles affect the kinetics features of Li${}_{2}$S nucleation and deposition, we employed a dimensionless current-time transient electrochemical deposition models based on the Scharifker-Hills and Beick-Fleischman equations ((S10)–(S14), Table S4). The 2DI and 2DP models denote an instantaneous (I) or progressive (P) two-dimensional nucleation process with adatom lattice incorporation governing the Li${}_{2}$S growth rate, whereas 3DI and 3DP imply the nucleation of a 3D hemispherical nucleus with ion diffusion regulating the growth rate [64,65]. These current-time transient are reported in Figure S10a and b for SN-rGO and SN-rGO/ZnS, respectively. The experimental curve of SN-rGO resembles a 2D-type nucleation and growth model, although there is no prevalence between the instantaneous or progressive process. Consequently, it can be hypothesized that a relatively large number of Li${}_{2}$S nucleation centers are formed on the SN-rGO surface and subsequently grow laterally, thus completing the 2D structure of the deposited Li${}_{2}$S. On the other hand, the SN-rGO/ZnS curve displays a trend much more similar to a 3DI model during the nucleation phase and the first part of the growth, while as the deposition reaction proceeds, the curve progressively settles to a hybrid 3DP/2D model. This trend suggests that the interfacial sites offered by ZnS nanoparticles might direct radial Li${}_{2}$S development, thus balancing surface lateral atomic diffusion and mass transfer in the electrolyte since ion diffusion primarily controls the growth rate in a 3D type deposition process. Therefore, the 3D deposition process of Li${}_{2}$S should be attributed to selective nucleation and growth near ZnS-rGO heterointerfaces with high activity [61], thus avoiding the total 2D passivation of the substrate and allowing a higher deposition capacity.

**Figure 5:**
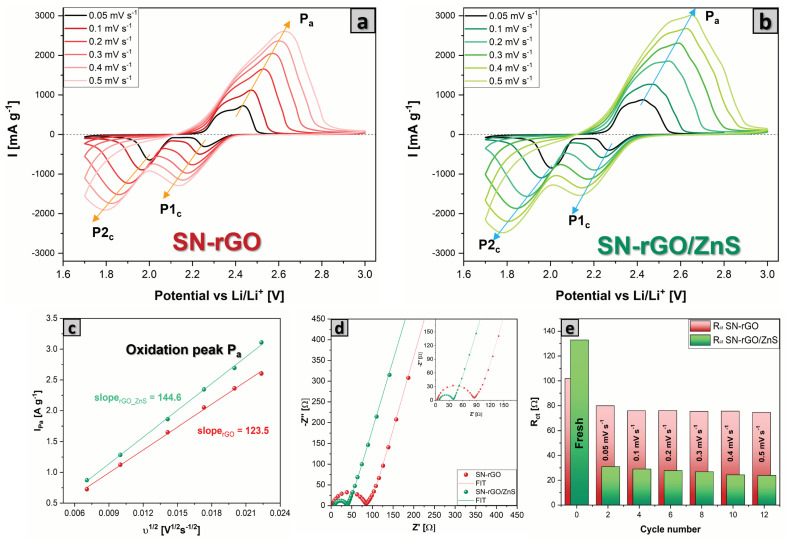
(**a**) CV of the SN-rGO/S${}_{8}$S-based cathode at different scan rate, (**b**) CV of the SN-rGO/ZnS/S${}_{8}$S-based cathode at different scan rate, (**c**) linear plot of the maximum current peak P${}_{a}$ for both SN-rGO/S${}_{8}$ and SN-rGO/ZnS/S${}_{8}$ vs. the square root of the scan rate, (**d**) EIS spectra of SN-rGO/S${}_{8}$S and SN-rGO/ZnS/S${}_{8}$ with the relative fitting, performed after the CV at 0.05 mVs${}^{-1}$, (**e**) comparison of the R${}_{c}t$ values for the two different materials recorded at 2.8 V after the second cycle of CV at each different scan rate.

In order to investigate the difference in electrochemical performance of Li–S cells based on different cathodes, coin-cell batteries with a sulfur loading of 1.1–1.3 mg cm${}^{-2}$ were assembled and electrochemically tested. Figure 5a,b shows the cyclic voltammetry (CV) results for Li-S batteries under the increasing sweeping rate from 0.05 mV s${}^{-1}$ to 0.5 mV s${}^{-1}$ for SN-rGO and SN-rGO/ZnS, respectively. Both samples exhibit two well-defined peaks located at 2.25 V (P1${}_{c}$) and 2.05 V (P2${}_{c}$) in the cathodic scan, which are ascribed to the reduction of S${}_{8}$ to high-order soluble Li${}_{2}$S${}_{x}$ (4 ≤x≤ 8) and followed by the reduction to low-order insoluble Li${}_{2}$S${}_{2}$/Li${}_{2}$S, respectively. Additionally, in the anodic scan, there is a single distinct peak around 2.4 V (denoted as P${}_{a}$), which, however, shows a shoulder at about 2.35 V for the SN-rGO and a second shoulder at about 2.48 V for the SN-rGO/ZnS. The presence of these sub-peaks highlights the gradual oxidation mechanism of Li${}_{2}$S to polysulphides and finally to S${}_{8}$ [66,67,68]. Furthermore, the positive catalytic activity played by ZnS nanoparticles was evaluated by the changes in onset potentials for the redox peaks in reduction and oxidation during the CV. Following a typical definition used in electrocatalysis, the onset potential was identified in correspondence with a current density of 10 μ A cm^2^ over the baseline current. A more detailed discussion about the determination of the onset potential is reported in Figures S11 and S12. Overall, the presence of ZnS decreases the onset potential of the anodic peak P${}_{a}$ while increasing those of the cathodic peaks in comparison with the pristine SN-rGO electrode (see Table S5 for the onset values), indicating faster kinetics thanks to the nanoparticles [67]. Moreover, by analyzing the dI/dV derivative curves obtained from the CVs (Figure S13), it was possible to identify the potential values at which the peaks P${}_{a}$, P1${}_{c}$S, and P2${}_{c}$ occur (reported in Table S5). Additionally, in this case, the trend previously shown by onset reaction potential is confirmed, with the SN-rGO/ZnS characterized by lower E P${}_{a}$ and higher E P1${}_{c}$S and P2${}_{c}$, thus making it possible to say that the SN-rGO/ZnS cell is less subject to polarization.

By increasing the scan rate of the CV, it was possible to investigate the lithium diffusion into the electrodes by using the Randles-Sevcik equation (Equation (2)), according to which the peak current (Ip) is linearly proportional to the square root of the scan rate, with a slope that is a function of the lithium diffusion coefficient in the material [69]. Figure 5c and Figure S14 show the linear plot of the peak current values vs. the square root of the scan rate for the three peaks present in the voltammograms (the higher the slope, the higher the diffusion coefficient). In Figure S15, the calculated lithium-ion diffusion coefficient values are plotted in function of the fraction of converted Li${}^{+}$ in the cathode [70]. The latter was estimated as follows: it was assumed that at the end of the cathodic scan, all the sulfur was converted into Li${}_{2}$S, so the fraction of lithium should be maximum and therefore, by convention, equal to 1. The fraction of lithium present in the cathode material was calculated by dividing the area under the maximum current of the peak considered by the area under the entire reduction branch for the P1${}_{c}$ and P2${}_{c}$ peaks, and the oxidation branch for the P${}_{a}$ peak. As reported in Figure S15, the lithium-ion diffusion coefficient is slightly higher in the case of SN-rGO/ZnS, thus confirming the beneficial role of the ZnS nanoparticles in the cathodic formulation.

The electrochemical impedance spectra (EIS) of the fresh cell and after each cycle of the CV at different scan rate was performed to get more information about the kinetics of the reactions occurring in our cells. As shown in Figure 5d and Figure S16, all the Nyquist plots basically consisted of one semicircle in high and medium frequencies, which represents the charge transfer resistance (R${}_{c}t$) and a straight line at lower frequencies, which is indicative of the lithium diffusion impedance (Z${}_{w}$) in the cathode. The equivalent circuit used to fit the data is reported in Figure S16. Interestingly, the fresh SN-rGO/ZnS cell exhibits a higher R${}_{c}t$ compared with the SN-rGO, probably due to the lower electronic conductivity of the ZnS nanoparticles compared with pure rGO. In contrast, already after the first cycle at 0.05 mVs${}^{-1}$ a trend reversal can be noted [70], with the R${}_{c}t$ of the SN-rGO becoming significantly higher than that of the SN-rGO/ZnS, as shown in Figure 5e. This inversion could be ascribed to the different deposition mechanisms of Li${}_{2}$S on the two materials, with the ZnS nanoparticles playing a pivotal role in mitigating the passivation of the cathode surface, which is consequently characterized by a lower resistance and a higher activity towards the LiPSs.

**Figure 6:**
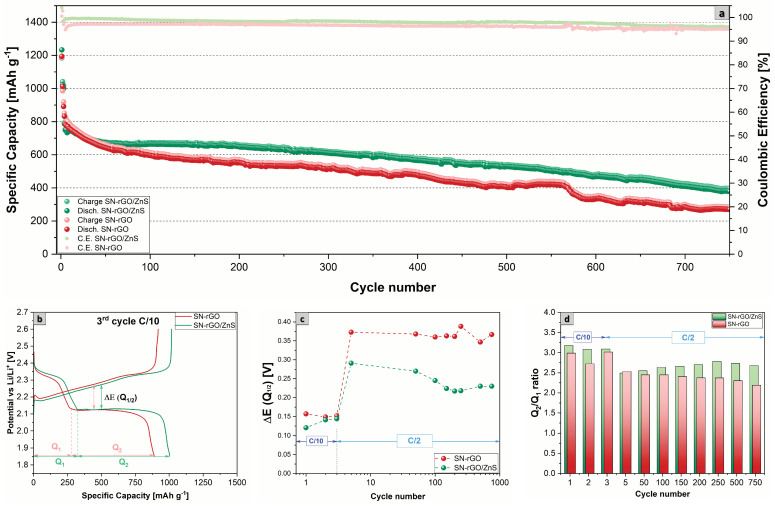
(**a**) Comparison of specific capacity over cycling at the current density of 0.5 C for 750 cycles of the SN-rGO/S${}_{8}$- and SN-rGO/ZnS/S${}_{8}$-based cathodes; (**b**) comparison of the charge-discharge voltage profiles of the third cycle at 0.1 C; (**c**) overpotential ΔE (Q${}_{1/2}$) between the lower discharge plateau and charge plateau over cycling; (**d**) comparison of the Q${}_{2}$/Q${}_{1}$ ratio values over cycling for the SN-rGO/S${}_{8}$- and SN-rGO/ZnS/S${}_{8}$-based cathodes.

To evaluate the electrochemical performance of our cathodes, we performed a long cycling in the potential window of 1.85–2.6 V vs. Li/Li${}^{+}$ under a current density of 0.5 C for 750 cycles. The long cycling was preceded by three formation cycles carried out at 0.1 C. The results are shown in Figure 6, and it is evident that the SN-rGO/ZnS/S${}_{8}$-based cathode can provide better performances over cycling: an initial discharge specific capacity of 786 mAh g${}^{-1}$ at the first cycle performed at 0.5 C was observed, which decreased to 379 mAh g${}^{-1}$ after 750 cycles (capacity retention of 48.2%), with a fading decay of 0.07% per cycle and a coulombic efficiency (CE) that ranged between 99.0% and 96.0%. In contrast, the SN-rGO/S${}_{8}$ delivered a higher initial discharge capacity (at the first cycle at 0.5 C) of 832 mAh g${}^{-1}$. However, the decay over cycling was more pronounced (0.09% per cycle with a final capacity retention of 32.5%) and a lower CE (97.1% at the first cycle at 0.5 C and 95.0% at the 750th cycle). The corresponding charge-discharge voltage profiles of each cell at the third cycle at 0.1 C are depicted in Figure 6b, from which it was possible to extract the polarization potential ΔE (Q${}_{1/2}$) between the second discharge plateau and the charge plateau. The values of the polarization for both SN-rGO/ZnS and SN-rGO over the 750 cycles are reported in Figure 6c, from which it is clear that at low C-rate the ΔE (Q${}_{1/2}$) is not particularly pronounced, while at 0.5 C the polarization is consistently higher for the SN-rGO/S${}_{8}$-based cathode. These results are consistent with the different onset potentials and the peak potential extracted from the CV measurements. Furthermore, in Figure S17 a magnification of the initial charge step of the third cycle at 0.1 C provides information about the activation energy required to start the oxidation process of Li${}_{2}$S: the potential jump exhibited by the SN-rGO is significantly higher than the one needed for the SN-rGO/ZnS (20.7 mV vs. 4.9 mV), a fact that confirms the hypothesis of the 2D passivation of graphene sheets by Li${}_{2}$S previously proposed [61]. Another critical parameter that can be extrapolated from the charge-discharge profiles is the ratio between the capacity associated with the two distinct plateaus, denoted as Q${}_{1}$ and Q${}_{2}$ in Figure 6b. In particular, the Q${}_{2}$/Q${}_{1}$ ratio is closely linked to the catalytic activity of the cathode material towards lithium polysulphides: slow kinetics during the liquid-to-solid conversion process occurring during the discharge and diffusion of LiPSs with consequent shuttle effect cause the capacity fading during Q${}_{2}$. Thus, the higher the ratio, the higher the catalytic ability of the cathodic formulation [71,72,73]. The values of the Q${}_{2}$/Q${}_{1}$ ratio for SN-rGO and SN-rGO/ZnS are reported in Figure 6d, with the latter constantly showing a higher percentage over the 750 cycles.

**Figure 7:**
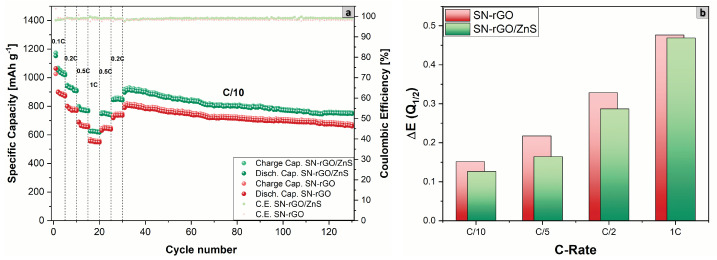
(**a**) Comparison of the rate capability performance at increasing C-rate of the SN-rGO/S${}_{8}$- and SN-rGO/ZnS/S_8_-based cathodes; (**b**) polarization between the low discharge plateau and charge plateau for the different cells vs. the applied C-rate.

Finally, the rate performance at increasing current density of the two different cathodic formulations was tested, as shown in Figure 7. Additionally, in this case, the SN-rGO/ZnS/S${}_{8}$-based cathode was able to deliver considerably higher specific discharge capacities with the increasing of the current density, as shown in Table S6, where also the reversible capacity for each scan rate is reported.

Moreover, as Figure 7b depicts, the polarization between the low discharge plateau and the charge plateau gradually increases for both SN-rGO and SN-rGO/ZnS (with the latter characterized by a significantly lower absolute value), with the difference between the two material almost disappearing at 1 C (a phenomenon that could indicate an intrinsic limitation of the material to cope with the increasing current).

## 4. Conclusions

This work reports a facile and fast microwave-assisted synthesis of a composite material SN-rGO/ZnS as a sulfur host for high-performance Li-S batteries. In particular, the proposed production method can significantly reduce the synthesis times compared to a classic hydrothermal process (30 min vs. several hours), ensuring the high reproducibility of the materials. The peculiarity of our cathode lies in the combination of a material with high electronic conductivity (sulfur-nitrogen doped rGO), which is required to have good use of sulfur, with a material (ZnS nanoparticles) that exhibits good affinity towards LiPSs and can catalyze the reaction of conversion from S${}_{8}$ to Li${}_{2}$S and vice versa during battery discharge/charge processes.

The chemical-physical characterization of our samples confirms, through XPS, FESEM, and XRD analysis, the heteroatomic (sulfur and nitrogen) doping process of graphene and the formation of ZnS nanoparticles on its surface. As reported in the literature, pyridinic nitrogen and ZnS nanoparticles are important factors since they are the main sites that can strongly interact with LiPSs.

The electrochemical characterization consisting of cyclic voltammetry, EIS, Li${}_{2}$S deposition test, long cycling at 0.5 C, and rate capability test (up to 1 C), highlighted, in the case of SN-rGO/ZnS, a lower polarization between the charge and discharge process, a more controlled Li${}_{2}$S nucleation and growth (with lower associated impedance), and higher specific capacities in both long cycling (capacity retention of 48.2% vs. 32.5% after 750 cycles) and rate capability test (648 vs. 517 mAh g${}^{-1}$ at 1 C). Overall, the microwave process exhibits excellent potential to synthesize highly reproducible materials with enhanced catalytic activity for Li-S battery cathodes.

## Data Availability

The data presented in this study are available upon request from the corresponding author.

## Supplementary Materials

The following supporting information can be downloaded at: https://www.mdpi.com/article/10.3390/nano13142149/s1, Figure S1: FESEM micrograph of the SN-rGO/ZnS sample, from which it is possible to evaluate the average dimensions of the ZnS nanoparticles. Figure S2: punctual EDS analysis of the nanoparticles attached to the surface of the SN-rGO/ZnS sample. Figure S3: EDS analysis of a larger area of the SN-rGO/ZnS sample. Figure S4: (a) XPS survey of SN-rGO and (b) SN-rGO/ZnS samples. Table S1: relative percentages of the element detected in the XPS surveys of SN-rGO and SN-rGO/ZnS. Figure S5: SN-rGO high resolution spectra of (a) C1s (b) S2p (c) N1s. Figure S6: high resolution spectra of Zn 2p for the SN-rGO/ZnS sample. Table S2: position [B.E.] and quantification [area %] of the peaks used to deconvolute the XPS data of SN-rGO and SN-rGO/ZnS. Figure S7: (a) adsorption-desorption isotherms of SN-rGO/S8 (b) adsorption-desorption isotherms of SN-rGO/ZnS/S8 sample. Figure S8: TGA analysis of SN-rGO and SN-rGO/ZnS samples. Figure S9: (a) cyclic voltammetry of free symmetrical cells using a solution of 1,2-dimethoxyethane (DME) and 1,3-dioxolane (DIOX) 1:1 (*v*/*v*) as electrolyte, performed in the −1~1 V window at the scan rate of 1 mV s${}^{-1}$ (b) cyclic voltammetry of free symmetrical cells with STD electrolyte 0.125 M using a solution of 1,2-dimethoxyethane (DME) and 1,3-dioxolane (DIOX) 1:1 (*v*/*v*) Li2S8 0.125 M, performed at 1 mVs${}^{-1}$. Table S3: reactions occurring at the working (WE) and counter electrode (CE) in the symmetric cells during the cyclic voltammetry in the ±1 V window. Table S4: list of equations used to model the different dimensionless current-time transient in Li2S deposition experiment. Figure S10: dimensionless current-time transient of (a) SN-rGO, (b) Sn-rGO/ZnS, obtained at 2.02 V in comparison with theoretical 2D and 3D models, where Im and tm are the peak current and the time needed to reach the peak current. Figure S11: (a) Differential CV curves of SN-rGO. The baseline voltage and current density are defined as the value before the redox peak, where the variation on current density is the smallest, namely dI/dV = 0. Baseline voltages are indicated by the dotted lines and the respective values for the cathodic scan in pink and for the anodic scan in red. (b) CV of the SN-rGO sample at the sweeping rate of 0.05 mV s${}^{-1}$ and the determination of the onset potential for the anodic peak and two cathodic peaks (Pa, P1_*c*_, P2_*c*_). According to a widely used definition in electrocatalysis, the onset potential occurs when the current density exceeds 10 µA cm${}^{-2}$ of the baseline current density (more specifically, 10 µA cm^−2^ more negative than the baseline current density for cathodic peaks or 10 µA cm${}^{-2}$ more positive than the baseline current density for anodic peaks). Figure S12: (a,b) Differential CV curves of SN-rGO/ZnS. Figure S13: (a) anodic scan and peak Pa of SN-rGO (b) cathodic scan and peaks P1_*c*_ and P2_*c*_ of SN-rGO performed at 0.05 mVs${}^{-1}$ (c) differential CV curves (dI/dV) of the anodic scan from which it is possible to individuate the exact position of the Pa peak (d) differential CV curves (dI/dV) of the cathodic scan from which it is possible to individuate the exact position of the P1_*c*_ and P2_*c*_ peaks for SN-rGO (e) anodic scan and peak Pa of SN-rGO/ZnS (b) cathodic scan and peaks P1_*c*_ and P2_*c*_ of SN-rGO/ZnS performed at 0.05 mVs${}^{-1}$ (c) differential CV curves (dI/dV) of the anodic scan from which it is possible to individuate the exact position of the Pa peak (d) differential CV curves (dI/dV) of the cathodic scan from which it is possible to individuate the exact position of the P1_*c*_ and P2_*c*_ peaks for SN-rGO/ZnS. Table S5: summary of the Eonset and and of the potentials at which the different current peaks (Pa, P1_*c*_ and P2_*c*_) are located for SN-rGO and SN-rGO/ZnS. Figure S14: (a) linear fitting of the reduction current peak P1_*c*_ values (occurring at 2.0–2.1 V) and (b) P2_*c*_ (occurring at 1.8–2.0 V) as function of the square root of the applied scan rate for SN-rGO (red) and SN-rGO/ZnS (green). Figure S15: comparison of the lithium-ion diffusion coefficient within the cathode material as function of the fraction of converted Li+ for SN-rGO and SN-rGO/ZnS. Figure S16: electrochemical impedence spectroscopy (EIS) performed on the coin cell assembled using lithium metal as anode and SN-rGO/S8 and SN-rGO/ZnS/S8 as cathode. The impeedence test was performed on (a) fresh cell (b) after the cyclic voltammetry at 0.1 mVs${}^{-1}$ at 2.8 V (c) afet the CV at 0.2 mVs${}^{-1}$ (d) after the CV at 0.3 mVs${}^{-1}$ (e) after the CV at 0.4 mVs${}^{-1}$ (f) after the CV at 0.5 mVs${}^{-1}$. At the center of the figure the equivalent circuit that was used to fit the EIS data is reported. Figure S17: magnification of the charge profile of the third cycle at 0.1 C which highlights the lower overpotential needed to activate the Li2S oxidation. Table S6: values of the rate capability performance of the SN-rGO and SN-rGO/ZnS. The reversible capacity of each scan rate was calculated dividing the discharge capacity of the first cycle (when the current density was gradually decreased after the five cycles at 1C) by the discharge capacity of the last (fifth) cycle at each current density when the current was gradually raised.
