# Supplementary material for: Reduced Graphene Oxide Embedded with ZnS Nanoparticles as Catalytic Cathodic Material for Li-S Batteries
Source: Nanomaterials (Basel). 2023 Jul 24;13(14):2149. doi: 10.3390/nano13142149 (PMC10384014; doi:10.3390/nano13142149)
Supplement: Supplementary file 1 [file nanomaterials-13-02149-s001.zip › nanomaterials-2503284-supplementary.pdf]

Supplementary Materials:

Reduced Graphene Oxide Embedded with ZnS Nanoparticles as Catalytic Cathodic Material for Li-S Batteries

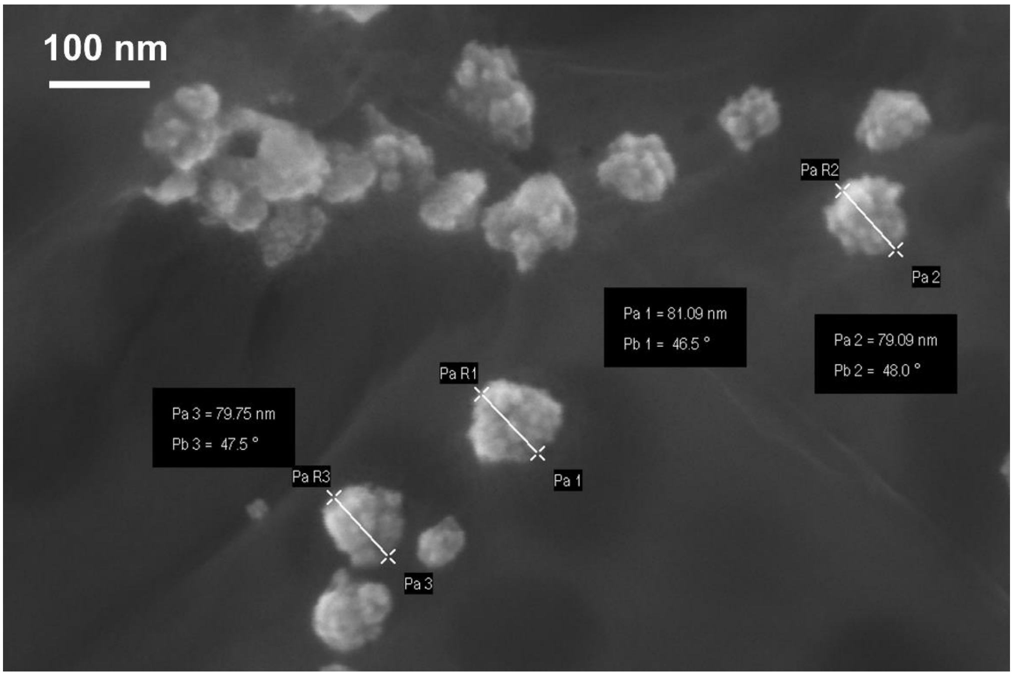

Figure S1: FESEM micrograph of the SN-rGO/ZnS sample, from which it is possible to evaluate the average dimensions of the ZnS nanoparticles.

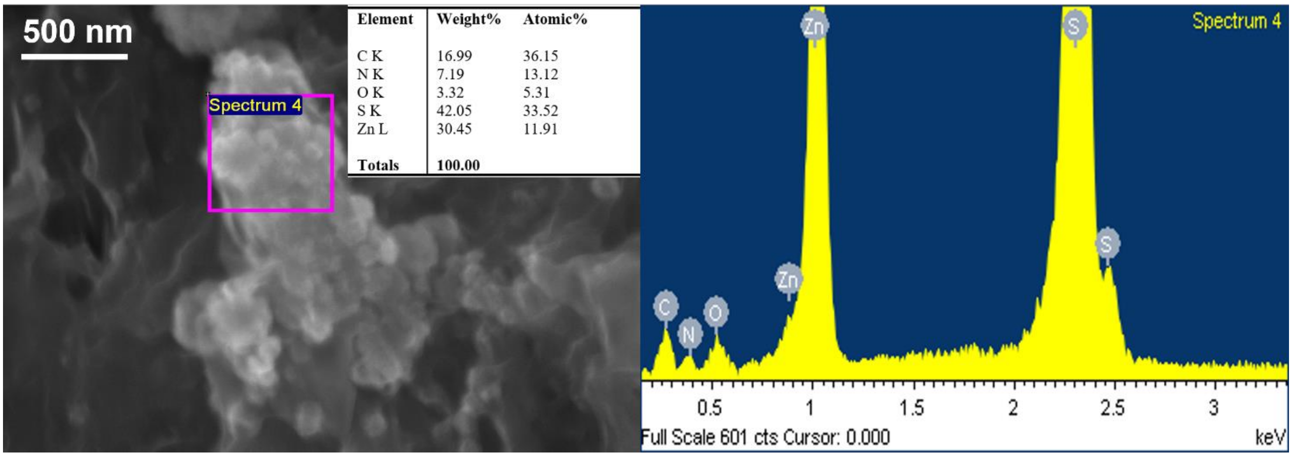

Element	Weight%	Atomic%
C K	16.99	36.15
N K	7.19	13.12
O K	3.32	5.31
S K	42.05	33.52
Zn L	30.45	11.91
Totals	100.00	

Figure S2: punctual EDS analysis of the nanoparticles attached to the surface of the SN-rGO/ZnS sample

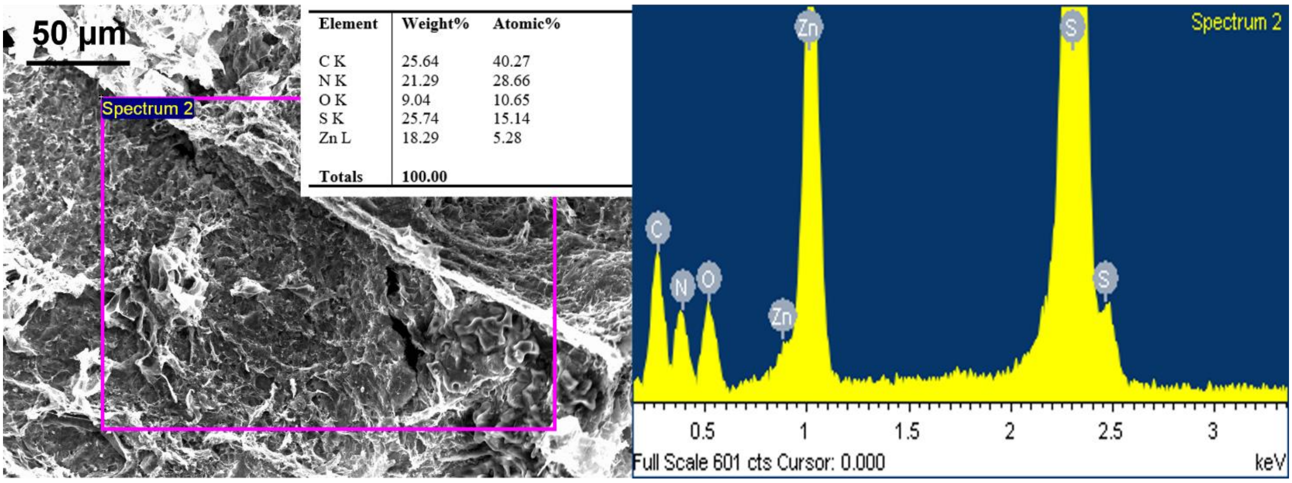

Figure S3: EDS analysis of a larger area of the SN-rGO/ZnS sample

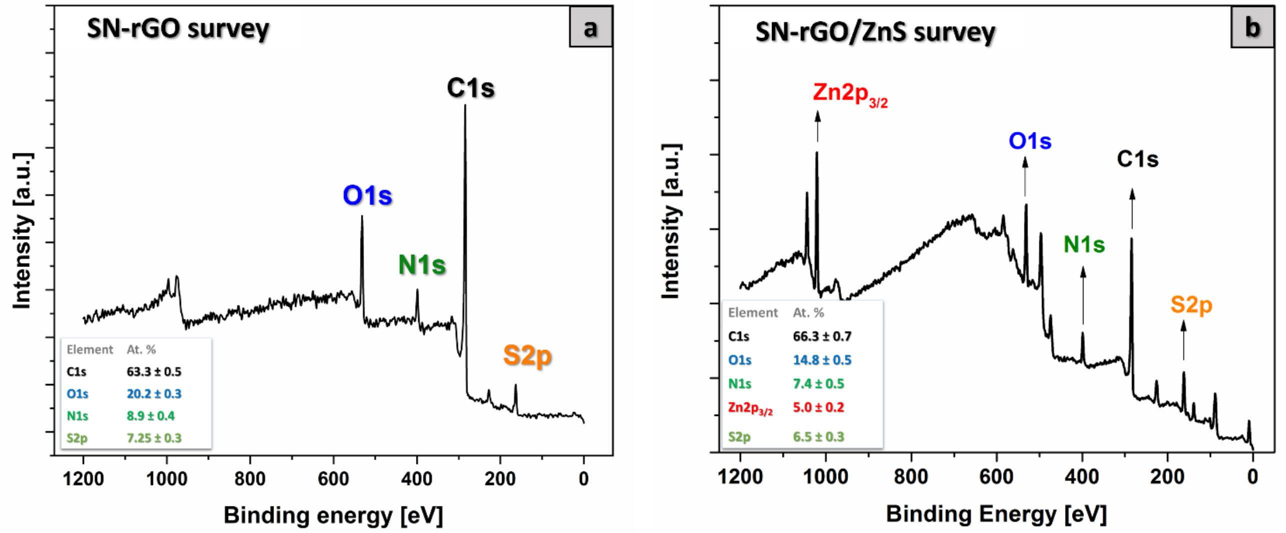

Figure S4: (a) XPS survey of SN-rGO and (b) SN-rGO/ZnS samples

Table S1: relative percentages of the element detected in the XPS surveys of SN-rGO and SN-rGO/ZnS

SAMPLE	C (at%)	O(at%)	N(at%)	S(at%)	Zn (at%)	C/O
**SN-rGO**	66.3 ± 0.5	20.2 ± 0.3	8.9 ± 0.4	7.25 ± 0.3	-	3.01
**SN-rGO/ZnS**	66.3 ± 0.7	14.8 ± 0.5	7.4 ± 0.5	6.5 ± 0.2	5.0 ± 0.3	4.62

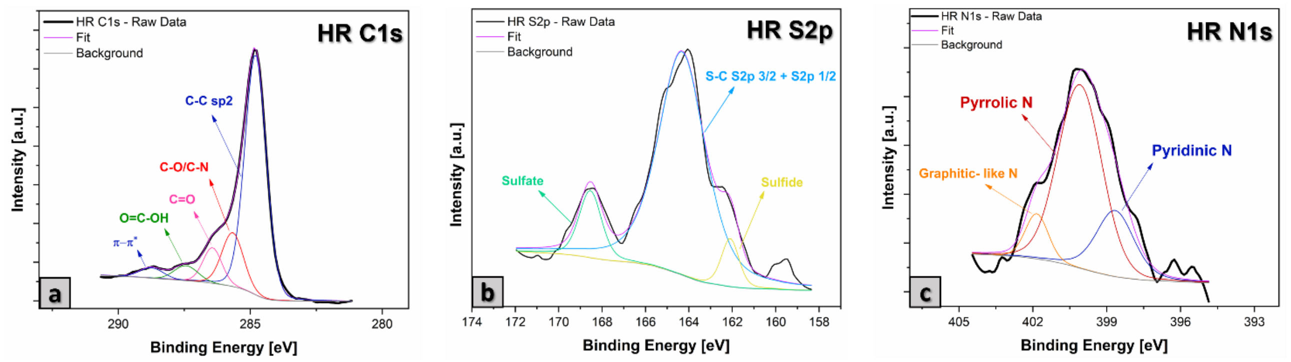

Figure S5: SN-rGO high resolution spectra of (a) C1s (b) S2p (c) N1s

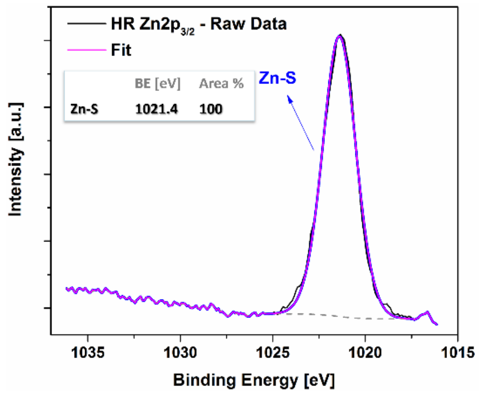

Figure S6: high resolution spectra of Zn 2p for the SN-rGO/ZnS sample

Table S2: position [B.E.] and quantification [area %] of the peaks used to deconvolute the XPS data of SN-rGO and SN-rGO/ZnS

		SN-RGO		SN-RGO/ZnS	
	**Specie**	**B.E. [eV]**	**Area [%]**	**B.E. [eV]**	**Area [%]**
**C1S**	C-C	284.8	68.17	284.5	84.4
	C-O/C-N	285.67	15.35	286.2	5.9
	C=O	286.43	8.8	287.4	3.7
	O=C-OH	287.43	4.48	288.9	4.7
	π- π*	288.74	3.12	291.3	1.3
**S2P**	S-Zn	-	-	160.8	42.8
	Sulfide	162.05	6.17	-	-
	-C-S-C	164.31	82.83	162.4	41.1
	-C=S-	-	-	163.6	16.1
	Sulfate	168.54	11	-	-
**N1S**	Pyridinic N	398.66	23.14	398.2	52.2
	Pyrrolic N	400.09	68.1	399.9	44.9
	Graphitic-like N	401.84	8.76	401.9	2.9

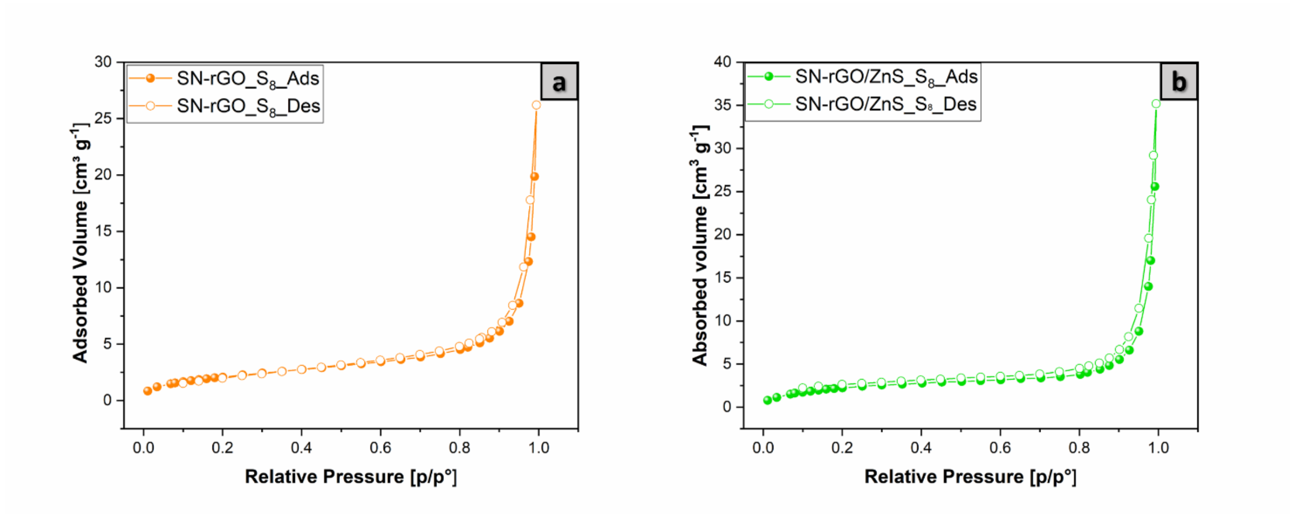

Figure S7: (a) adsorption-desorption isotherms of SN-rGO/S_8 (b) adsorption-desorption isotherms of SN-rGO/ZnS/S_8 sample

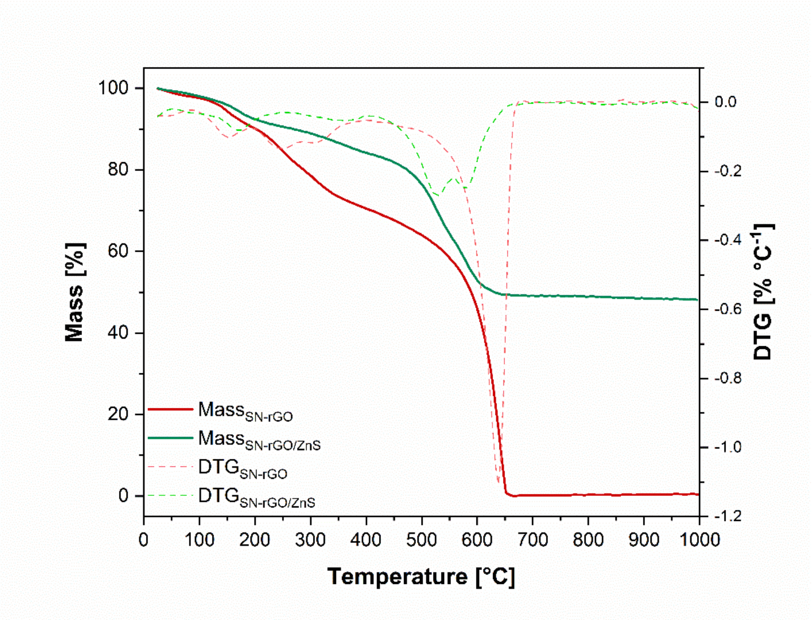

Figure S8: TGA analysis of SN-rGO and SN-rGO/ZnS samples

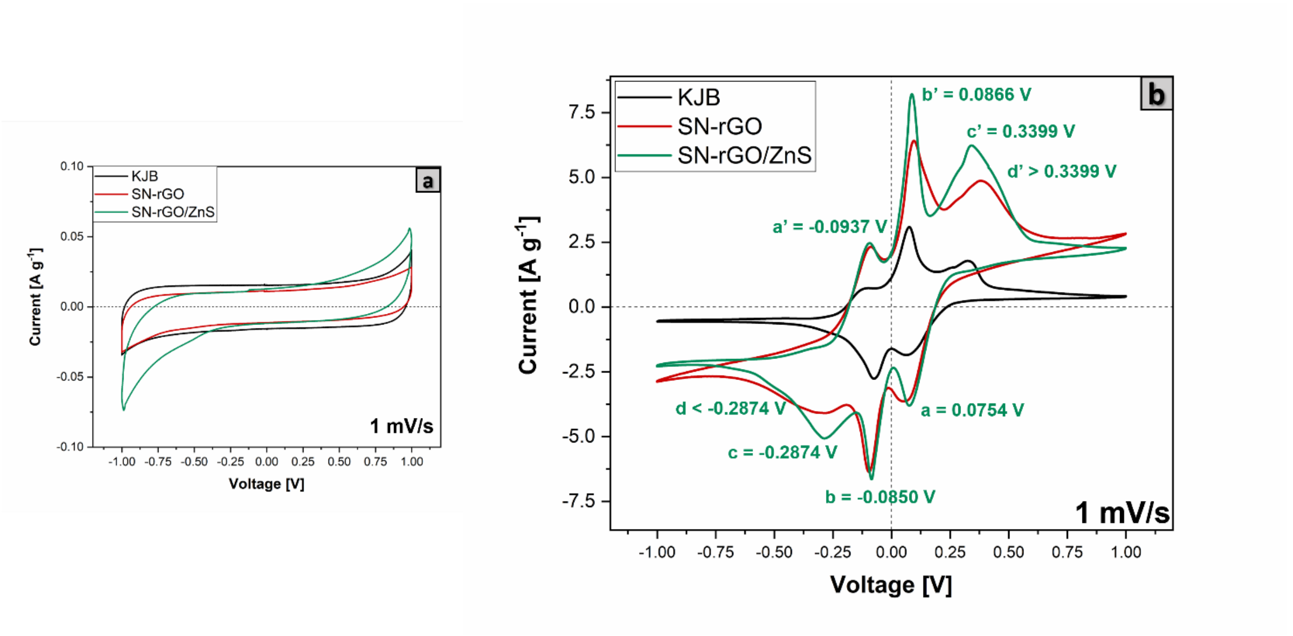

Figure S9: (a) cyclic voltammetry of free symmetrical cells using a solution of 1,2-dimethoxyethane (DME) and 1,3-dioxolane (DIOX) 1:1 (v/v) as electrolyte, performed in the -1 ~ 1 V window at the scan rate of 1 mV s^{-1} (b) cyclic voltammetry of free symmetrical cells with STD electrolyte 0.125M using a solution of 1,2-dimethoxyethane (DME) and 1,3-dioxolane (DIOX) 1:1 (v/v) Li_2S_8 0.125M, performed at 1 mVs^{-1}

Table S3: reactions occurring at the working (WE) and counter electrode (CE) in the symmetric cells during the cyclic voltammetry in the ± 1 V window. For the a′, b′, c′, d′ peaks the reactions must be reversed between the WE and CE. The peaks d and d' cannot be detected clearly from the voltammograms in Figure 4b and S9b, but their presence as shoulder of the peaks c and c′ is justified by the widening of those peaks.

Peak	Voltage [V]	Reaction
a	0.0754	**WE:** $Li_2S_8 + \frac{2}{3}Li^+ + \frac{2}{3}e^- \rightarrow \frac{4}{3}Li^+ \; Li_2S_6$ **(S1)**
		**CE:** $8Li_2S - 8Li^+ - 8e^- \rightarrow 4Li_2S_8$ **(S2)**
b	-0.0850	**WE:** $\frac{4}{3}Li_2S_6 + \frac{4}{3}Li^+ + \frac{4}{3}e^- \rightarrow 2Li_2S_4$ **(S3)**
		**CE:** $4Li_2S_2 - 4Li^+ - 4e^- \rightarrow 2Li_2S_4$ **(S5)**
c	-0.2874	**WE:** $2Li_2S_4 + 4Li^+ + 4e^- \rightarrow 2Li_2S_2$ **(S6)**
		**CE:** $2Li_2S_4 - \frac{4}{3}Li^+ - \frac{4}{3}e^- \rightarrow \frac{4}{3}Li_2S_6$ **(S7)**
d	< -0.2874	**WE:** $4Li_2S_2 + 8Li^+ + 8e^- \rightarrow 8Li_2S$ **(S8)**
		**CE:** $\frac{4}{3}Li_2S_6 - \frac{2}{3}Li^+ - \frac{2}{3}e^- \rightarrow Li_2S_8$ **(S9)**

Table S4: list of equations used to model the different dimensionless current-time transient in Li_2S deposition experiment.

INSTANTANEOUS NUCLEATION (2DI) – EQUATION (S10)	PROGRESSIVE NUCLEATION (2DP) – EQUATION (S11)	INSTANTANEOUS NUCLEATION (3DI) – EQUATION (S12)	PROGRESSIVE NUCLEATION (3DP)- EQUATION (S13)
$\frac{j}{j_m} = \left(\frac{t}{t_m}\right)\left\{exp\left[\frac{t_m - t_m^2}{2t_m^2}\right]\right\}$	$\frac{j}{j_m} = \left(\frac{t}{t_m}\right)\left\{exp\left[\frac{-2(t^3 - t_m^3)}{3t_m^3}\right]\right\}^2$	$\frac{j}{j_m} = \left(\frac{1.9542}{t/t_m}\right)^{0.5}\left\{1 - exp\left[1.2564\left(\frac{t}{t_m}\right)\right]\right\}$	$\frac{j}{j_m} = \left(\frac{1.2254}{t/t_m}\right)^{0.5}\left\{1 - exp\left[2.3367\left(\frac{t}{t_m}\right)^2\right]\right\}$

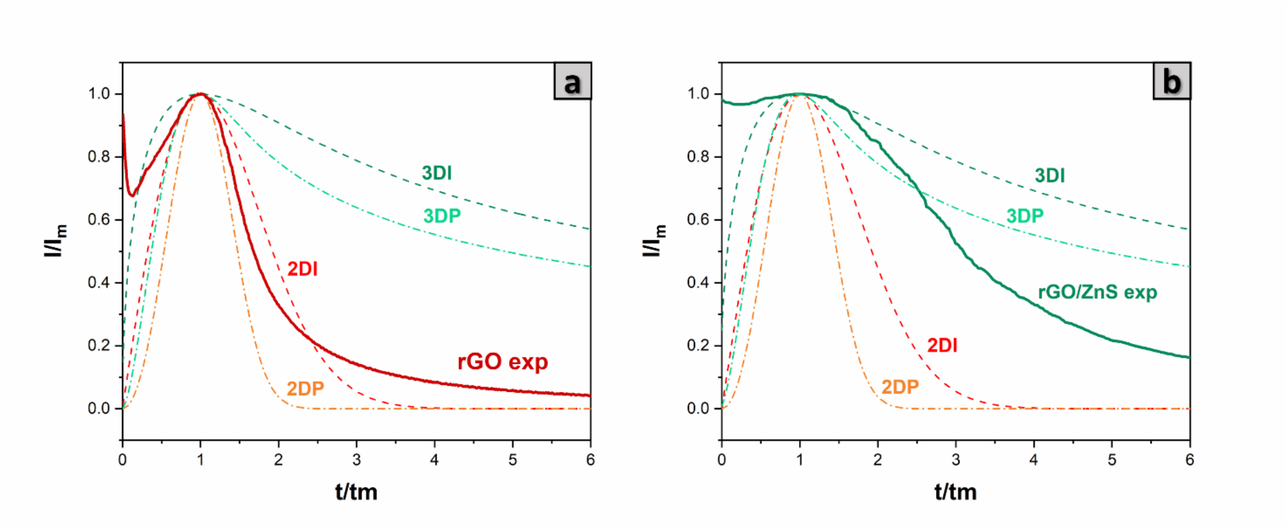

Figure S10: dimensionless current-time transient of (a) SN-rGO, (b) Sn-rGO/ZnS, obtained at 2.02V in comparison with theoretical 2D and 3D models, where I_m and t_m are the peak current and the time needed to reach the peak current.

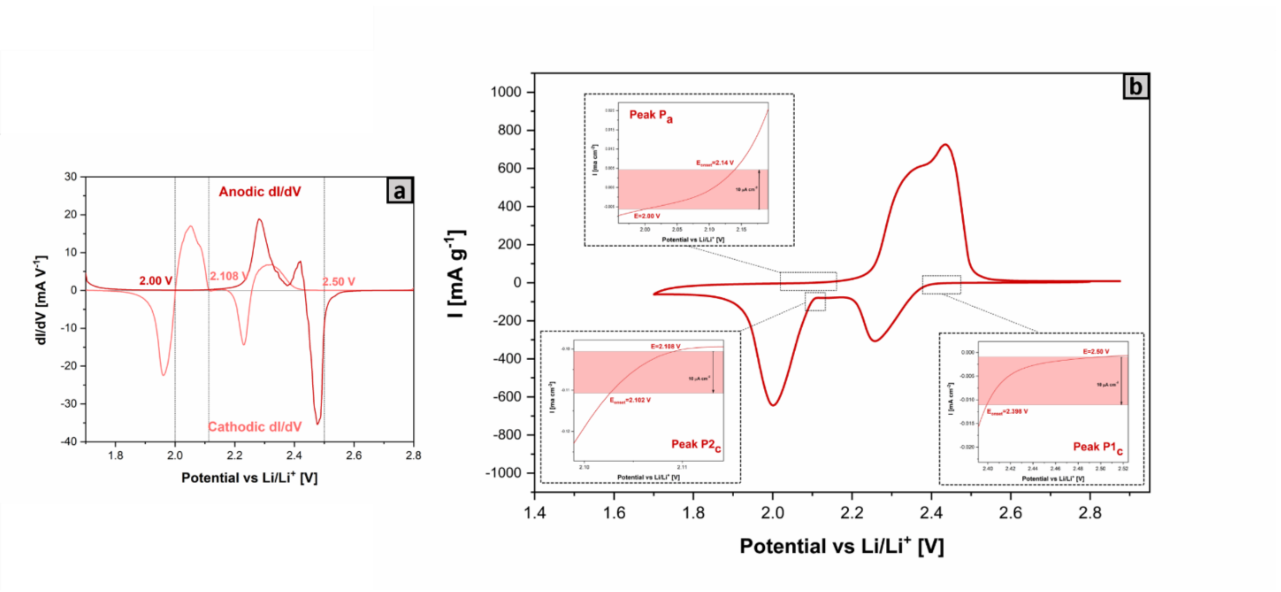

Figure S11: (a) Differential CV curves of SN-rGO. The baseline voltage and current density are defined as the value before the redox peak, where the variation on current density is the smallest, namely dI/dV= 0. Baseline voltages are indicated by the dotted lines and the respective values for the cathodic scan in pink and for the anodic scan in red. (b) CV of the SN-rGO sample at the sweeping rate of 0.05 mV s^{-1} and the determination of the onset potential for the anodic peak and two cathodic peaks (P_a, $P1_c$, $P2_c$). According to a widely used

definition in electrocatalysis, the onset potential occurs when the current density exceeds 10 μA cm^{-2} of the baseline current density (more specifically, 10 μA cm^{-2} more negative than the baseline current density for cathodic peaks or 10 μA cm^{-2} more positive than the baseline current density for anodic peaks).

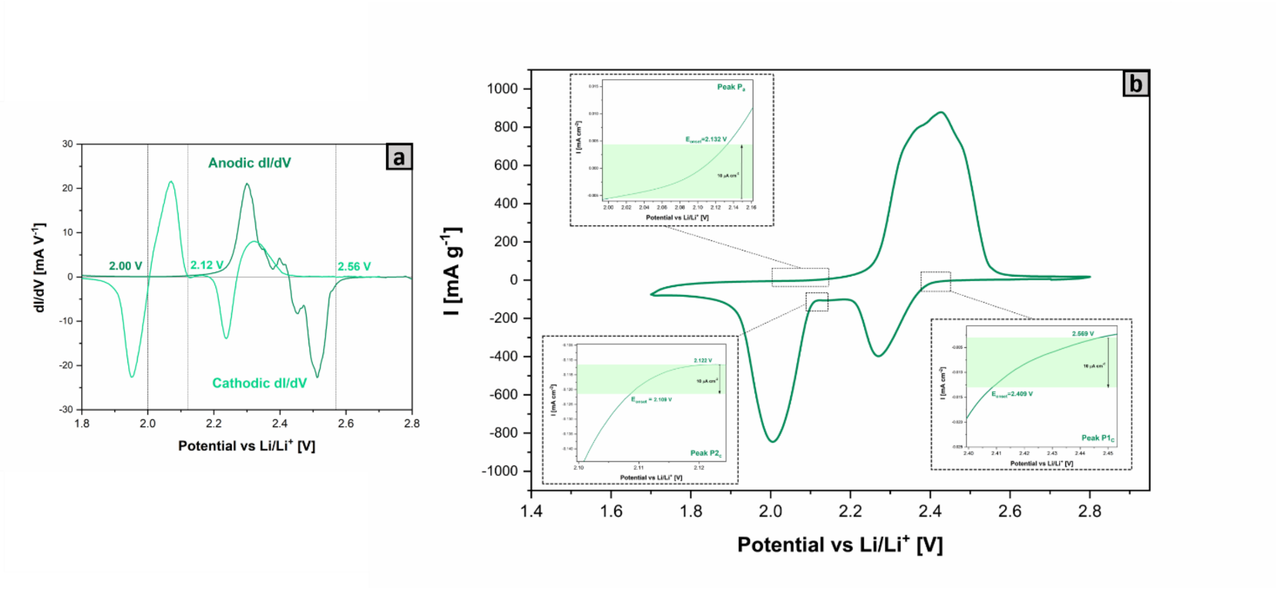

Figure S12: (a) (b) Differential CV curves of SN-rGO/ZnS. The baseline voltage and current density are defined as the value before the redox peak, where the variation on current density is the smallest, namely dI/dV= 0. Baseline voltages are indicated by the dotted lines and the respective values for the cathodic scan in light green and for the anodic scan in dark green. (b) CV of the SN-rGO/ZnS sample at the sweeping rate of 0.05 mV s^{-1} and the determination of the onset potential for the anodic peak and two cathodic peaks (P$_a$, P1$_c$, P2$_c$).

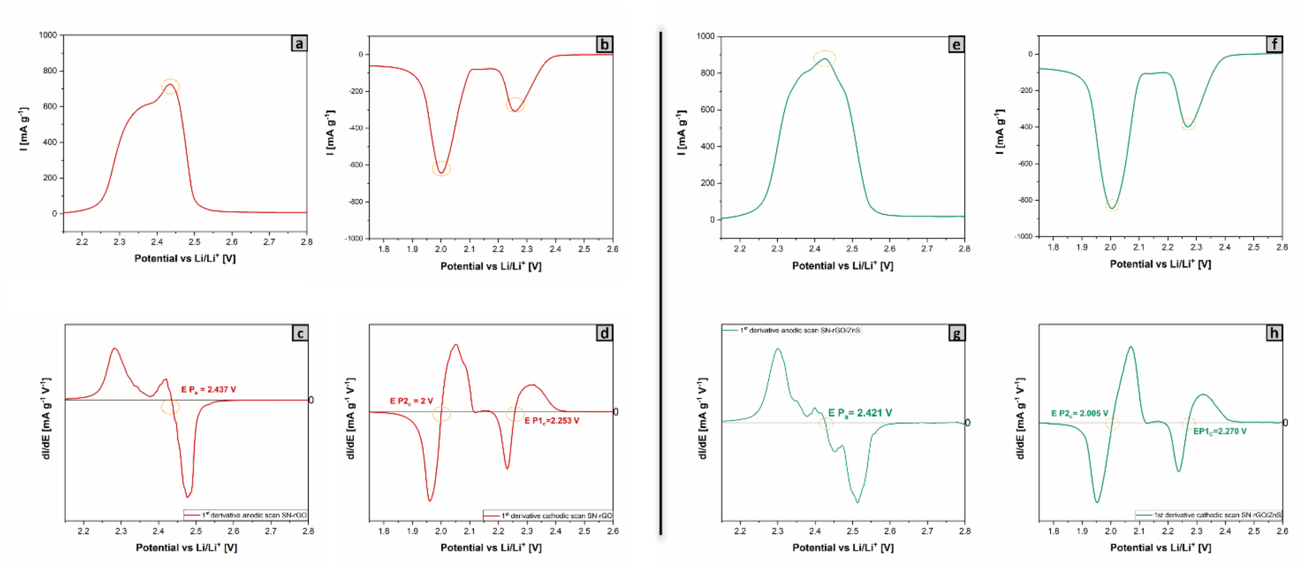

Figure S13: (a) anodic scan and peak P$_a$ of SN-rGO (b) cathodic scan and peaks P1$_c$ and P2$_c$ of SN-rGO performed at 0.05 mVs^{-1} (c) differential CV curves (dI/dV) of the anodic scan from which it is possible to individuate the exact position of the P$_a$ peak (d) differential CV curves (dI/dV) of the cathodic scan from which it is possible to individuate the exact position of the P1$_c$ and P2$_c$ peaks for SN-rGO (e) anodic scan and peak P$_a$ of SN-rGO/ZnS (b) cathodic scan and peaks P1$_c$ and P2$_c$ of SN-rGO/ZnS performed at 0.05 mVs^{-1} (c) differential CV curves (dI/dV) of the anodic scan from which it is possible to individuate the exact position of the P$_a$ peak (d) differential CV curves (dI/dV) of the cathodic scan from which it is possible to individuate the exact position of the P1$_c$ and P2$_c$ peaks for SN-rGO/ZnS.

Table S5: summary of the E_{onset} and and of the potentials at which the different current peaks (P_a, $P1_c$ and $P2_c$) are located for SN-rGO and SN-rGO/ZnS.

Sample	E_{onset} P_a [V]	E_{onset} $P1_c$ [V]	E_{onset} $P2_c$ [V]	E P_a [V]	E $P1_c$ [V]	E $P2_c$ [V]
**SN-rGO**	2.14	2.376	2.102	2.437	2.253	2.000
**SN-rGO/ZnS**	2.132	2.409	2.109	2.421	2.270	2.005

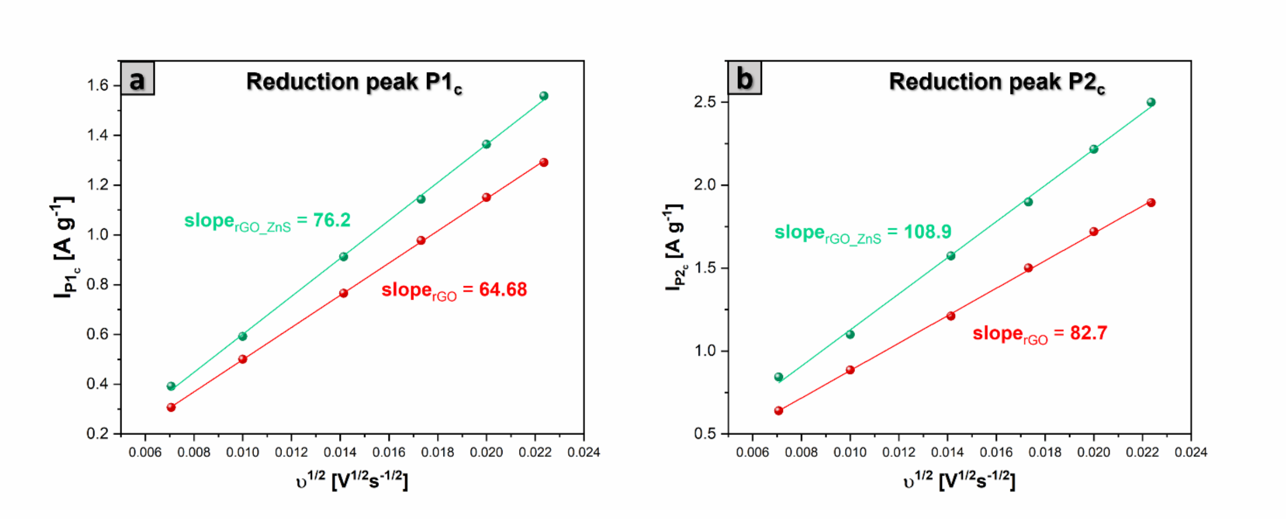

Figure S13: (a) linear fitting of the reduction current peak $P1_c$ values (occurring at 2.0-2.1 V) and (b) P2c (occurring at 1.8-2.0 V) as function of the square root of the applied scan rate for SN-rGO (red) and SN-rGO/ZnS (green).

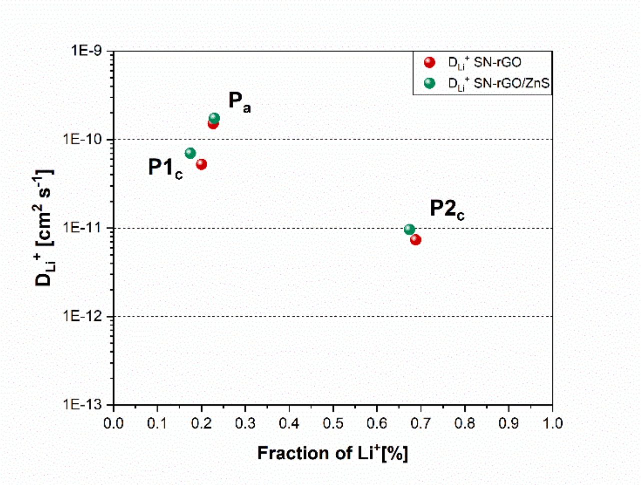

Figure S14: comparison of the lithium-ion diffusion coefficient within the cathode material as function of the fraction of converted Li^+ for SN-rGO and SN-rGO/ZnS.

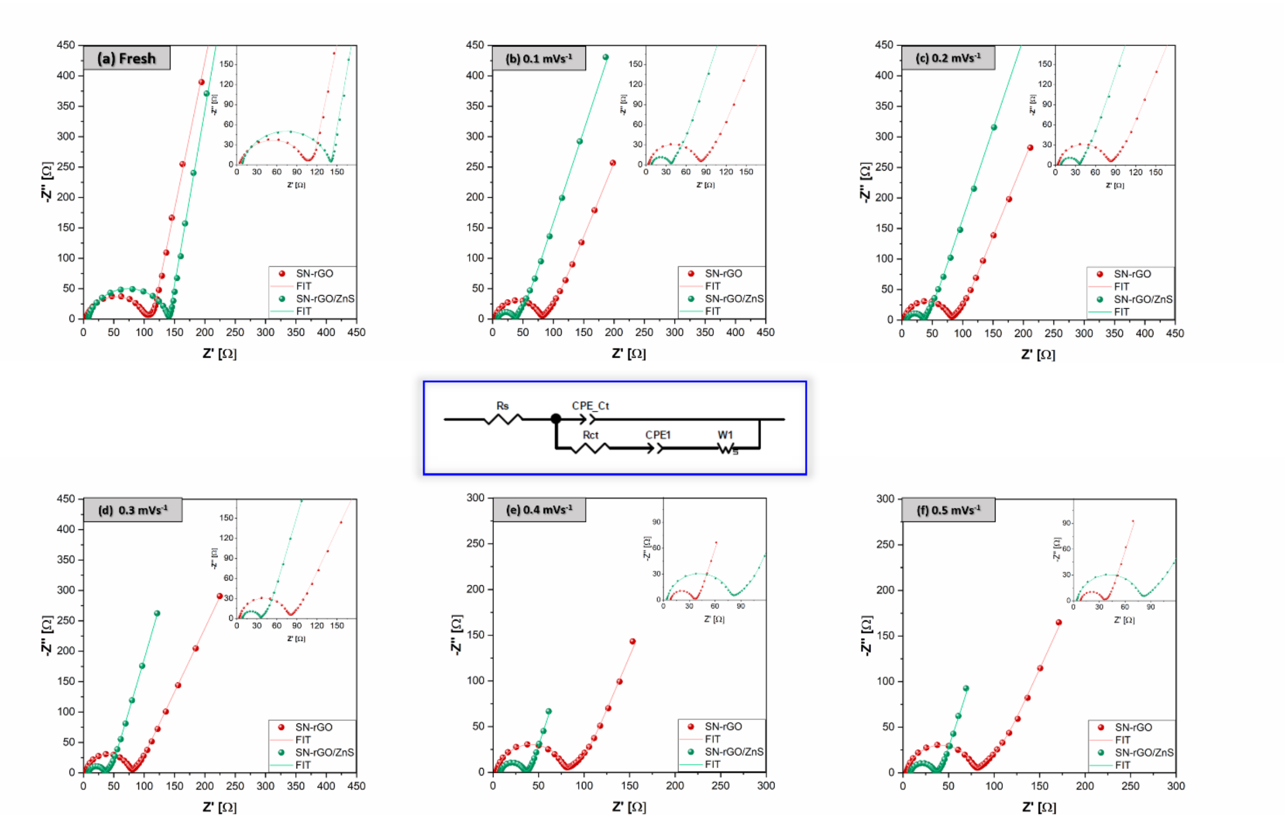

Figure S15: electrochemical impedence spectroscopy (EIS) performed on the coin cell assembled using lithium metal as anode and SN-rGO/S_8 and SN-rGO/ZnS/S_8 as cathode. The impeedence test was performed on (a) fresh cell (b) after the cyclic voltammetry at 0.1 mVs^{-1} at 2.8 V (c) afet the CV at 0.2 mVs^{-1} (d) after the CV at 0.3 mVs^{-1} (e) after the CV at 0.4 mVs^{-1} (f) after the CV at 0.5 mVs^{-1}. At the center of the figure the equivalent circuit that was used to fit the EIS data is reported.

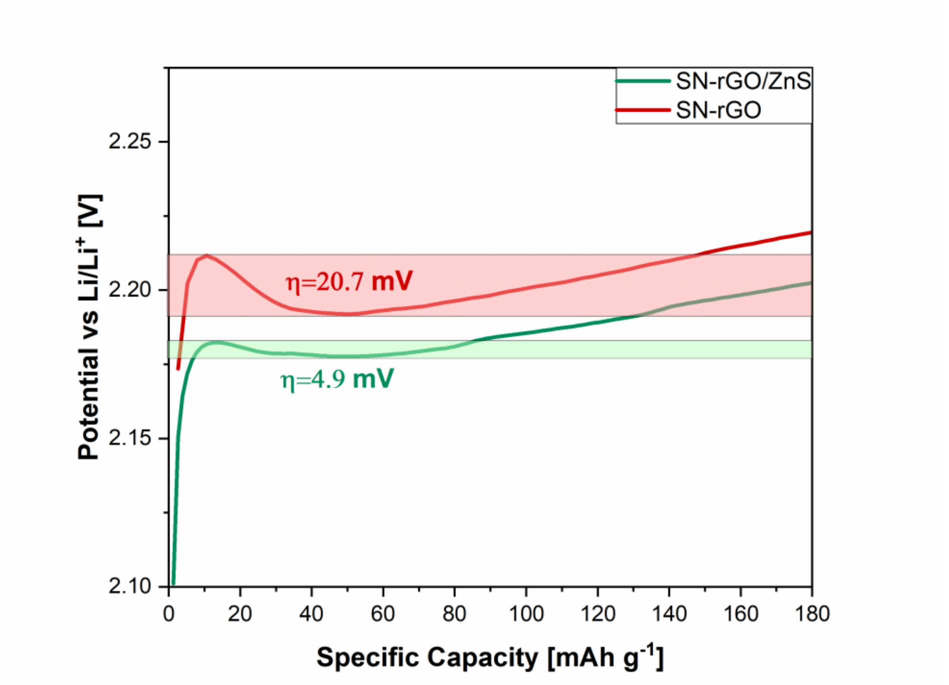

Figure S16: magnification of the charge profile of the third cycle at 0.1C which highlights the lower overpotential needed to activate the Li_2S oxidation.

Table S6: values of the rate capability performance of the SN-rGO and SN-rGO/ZnS. The reversible capacity of each scan rate was calculated dividing the discharge capacity of the first cycle (when the current density was gradually decreased after the five cycles at 1C) by the discharge capacity of the last (fifth) cycle at each current density when the current was gradually raised.

**Sample**	**0.1C [mAh]**	**0.5C**	**0.2C**	**1C**	**Reversible capacity 0.2C [%]**	**Reversible capacity 0.5C [%]**	**Reversible capacity 0.1C [%]**
**SN-rGO/S_8**	872	769	653	547	96.1	93.1	90.0
**SN-rGO/ZnS/S_8**	1017	903	766	613	97.3	93.6	90.2
